# Inference and forecasting phase shift regime of COVID-19 sub-lineages with a Markov-switching model

**DOI:** 10.1128/spectrum.01669-23

**Published:** 2023-10-09

**Authors:** Eul Noh, Jinwook Hong, Joonkyung Yoo, Jaehun Jung

**Affiliations:** 1 Freddie Mac, Tysons Corner, Virginia, USA; 2 Artificial Intelligence and Big-Data Convergence Center, Gil Medical Center, Gachon University College of Medicine, Incheon, South Korea; 3 Department of Economics, Rutgers University--New Brunswick, New Brunswick, New Jersey, USA; 4 Department of Preventive Medicine, Gachon University College of Medicine, Incheon, South Korea; IrsiCaixa Institut de Recerca de la Sida, Badalona, Barcelona, Spain

**Keywords:** SARS-CoV-2, COVID-19, variant, forecasting, Markov-switching model, regime switch, phase shift, volatility

## Abstract

The occurrences and domination of severe acute respiratory syndrome coronavirus 2 (SARS-CoV-2) variants are still crucial factors for determining the coronavirus disease 19 (COVID-19) policies. We collected weekly Phylogenetic Assignment of Named Global Outbreak sub-lineages, naming genetically distinct lineages of SARS-CoV-2, including variants of concern, in the United Kingdom, South Africa, South Korea, Denmark, Germany, the United States, and worldwide. This study included 12,296,756 samples of the max share of the sub-lineages from the 33rd week of 2020 to the 40th week of 2022. This study conducted a two-state Markov-switching model to estimate the probability of the phase shift state and predicted the probability of each regime with the Hamilton filter and Kim’s smoothing algorithm. We discovered different weekly patterns based on dominant SARS-CoV-2 variants in target area. Due to differences in containment policies and outbreak waves, we observed a time lag in dominant variants in these countries. Using the inferred probability of the phase shift regime for forecasting, it showed significant probabilities that the stable phase will still be stable in the next week. It also showed significant probabilities that the unstable phase will still be unstable in the next week. Our findings present the probability of observing the phase shift regime each week. Until a new SARS-CoV-2 variant occurs, the regime tended to stay with a low probability of phase shift regime. When a new SARS-CoV-2 variant would occur, the regime would immediately react and change the probability. We propose the Markov-switching model to determine COVID-19 policies and predict SARS-CoV-2 variants.

**IMPORTANCE:**

Using regime-switching models, we attempted to determine whether there is a link between changes in severe acute respiratory syndrome coronavirus 2 (SARS-Cov-2) variants and infection waves, as well as forecasting new SARS-Cov-2 variants. We believe that our study makes a significant contribution to the field because it proposes a new approach for forecasting the ongoing pandemic, and the spread of other infectious diseases, using a statistical model which incorporates unpredictable factors such as human behavior, political factors, and cultural beliefs.

## INTRODUCTION

Since the emergence of the severe acute respiratory syndrome coronavirus 2 (SARS-CoV-2) in December 2019 in China’s Wuhan city, the fear of escalating SARS-CoV-2 has continued globally (1). Prior research showed that SARS-Cov-2 had developed many mutations, especially since there are two major lineages, namely L and S (2). Because of mutations, several countries have experienced infection waves that were replaced by the Alpha (B.1.1.7) variant of concern, then by Delta (B.1.617.2), and a fourth wave consisting of Omicron (B.1.1.529) for more than 2 years into the coronavirus disease 19 (COVID-19) pandemic (3). Hence, it is important to prevent epidemics and to predict the new coronavirus variants on the spread of the pandemic. COVID-19 vaccines have also been introduced to reduce virus transmission, an effective critical tool for preventing disease (4). Despite this, variants have continued to evolve. In addition, increasing vaccine breakthrough cases or vaccine hesitancy made pandemic prediction difficult (5).

Early studies have used several methods to forecast infectious diseases, including statistical methods, the susceptible-exposed-infectious-recovered (SEIR) model (6), the autoregressive integrated moving average (ARIMA) model (7), the long short-term memory model (8), and the Markov-switching autoregressive model (9). The SEIR model predicted the development tendency of COVID-19 and that the mortality rate was fixed during the study period. Moreover, even though the ARIMA model holds a linear relationship between past phenomena and the future, the application of this approach is limited to short-term prediction and does not apply to the long-term prediction of COVID-19. Due to the political, economic, and social issues related to the pandemic uncertainty in each country, existing forecasting models have been limited in proving their predictions’ accuracy. Furthermore, traditional compartment models struggle to forecast new mutations and their potential impact on infection spread. Given the importance of anticipating novel mutations and their effects on trend curves, our model seeks to provide quantitative predictions, as opposed to scenario-based approaches. While molecular biology has attempted to predict new mutations, no successful method has been developed thus far. Therefore, we still needed a developed way of forecasting outbreaks of the new coronavirus variants because the evolution of COVID-19 variants differed across countries depending on socio-economic characteristics (10).

This study employs a developed method, the Markov-switching model, to overcome prediction errors in previous studies. The Markov-switching model allows for calculating the conditional probability of the phase shift regime each week, given the history of available information without a priori knowledge about the timing of the regime changes (11). The model assumed the autoregressive structure of the max share and the unobserved state variable in the volatility of the max share variable. The regime probabilities were computed with a filtering algorithm. Additionally, since the model is based on a stochastic process, it could make short-term and long-term predictions of a variant emergence in the COVID-19 pandemic from variances of the errors (12). Specifically, the Markov-switching model allowed us to predict the probability of a regime in which a new variant changes the existing dominance sub-lineage by forecasting the probability of the high-volatility regime. Note that the conventional time-varying volatility models with continuous conditional variance such as generalized autoregressive conditional heteroskedasticity (GARCH) do not provide the information about the discrete state of the volatility. Additionally, our Markov-switching model shows slightly better measures of the model fitness for the max share variable. We present the comparison of the fitness measures for the max share variable including mean absolute percentage error, mean squared error, and root mean squared error between the Markov-switching model and GARCH (Supplementary Material S1).

This study suggests a Markov-switching model that infers and forecasts the probability of observing periods in which an existing dominant variance of SARS-CoV-2 and a new variance actively compete, and the rank of the two variances is changed. We identified the SARS-CoV-2 variants using PANGO sub-lineage data and estimated the model for six countries—Denmark, Germany, Korea, South Africa, the United Kingdom, the United States—and the rest of the world.

## MATERIALS AND METHODS

### Data collection

We estimated the regime changes among the sub-lineages (phase shift or stable dominance) in six countries (Denmark, Germany, Korea, South Africa, the United Kingdom, and the United States) and worldwide. Considering the lineage list, we obtained all period data of sub-lineages of PANGO, which had spread from covSPECTRUM (13). We collected weekly sub-lineages in which the proportion is greater or equal to 0.1%, except Omicron PANGO, from the 2nd week of 2020 to the 44th week of 2022 of six regions except for Korea. For Korea, we collected the same ones in which the proportion is greater or equal to 0.02%. The reason being because Korean government’s intense quarantine policy led to the number of sequences collected being too low to analyze with a 0.1% hurdle of sampling. Therefore, each sub-lineage was used from the 32nd week of 2020 to the 40th week of 2022. For Omicron PANGO in all regions, we collected and used all data of its sub-lineage of all regions we investigated. However, since some countries had missing and uneven data, we used Omicron PANGO as B.1.1.529, BA.1*, BA.2*, BA.2.75*(Centaurus), BA.3*, BA.4*, and BA.5*. Supplementary Material S2 contains the number of samples for PANGO lists, and Supplementary Material S3 contains the sequence number of PANGO sub-lineages across countries in the period.

### Study design

In our study, the main variable of interest $y_{t}$ is defined by the share of largest sub-lineage in each week, *t*. In summary, $y_{t}=max\left(y_{1,t},\cdots ,y_{n,t}\right)$ , where $y_{i,t}$ denotes the share of i th sub-lineage out of n in week t , satisfying $\sum_{i=1}^{n}y_{i,t}=1$ for each t=1,2,⋯T . Our data indicate that when a new dominating sub-lineage emerges, it quickly substitutes the existing dominating sub-lineage. In other words, in the phase-shifting periods, the conditional volatility of $y_{t}$ (i.e., the variance of $\epsilon_{t})$ ) was significantly higher than in the stable dominance periods. The main purpose of our model is to infer the probability of the phase-shifting regime in each week and forecast the probability of observing the phase shift in the future by using the time-varying volatility of the max share variable $y_{t}$ . To distinguish the high- and low-volatility regimes, we employed autoregressive model of $y_{t}$ with conditional variance following a two-state Markov-switching process $S_{t}$ as follows:


(1) $$y_{t}=\mu +\sum_{l=1}^{p}\phi_{l}y_{t-l}+\sum_{l=1}^{q}\delta_{l}\epsilon_{t-l}+\epsilon_{t}$$

(2) $$\epsilon_{t}∼N\left(0,\sigma_{S_{t}}^{2}\right),\;\;\sigma_{0}\leq \sigma_{1}$$

(3) $$\mathrm{Pr}\left(S_{t}=j|S_{t-1}\right)=p_{ij}\;\text{for }i,j=0,1$$

$p_{ij}$ is the transition probability of the regimes satisfying $\sum_{j=0}^{1}p_{ij}=1$ for i=0 and 1. We interpreted high-volatility state ( $S_{t}=1$ ) as the phase shift regime and the low-volatility state ( $S_{t}=0$ ) as the stable dominance regime.

### Statistical analysis

Since we did not have a priori information about the timing of the phase shift, the probability of $S_{t}=1$ was estimated with given observable sub-lineage information. For the optimal value of the parameters for estimating the probability of the phase shift state, we used the maximum likelihood estimation (MLE) approach. Specifically, we maximized the value of log likelihood values computed with Hamilton filter (14) corresponding to the value of the parameter set by adjusting the parameter values using the Newton-Raphson algorithm. After the MLE estimation, we also estimated forecasting the probability of each regime with Hamilton filter and Kim’s smoothing algorithm (15). Forecasting the out-of-sample probability of the phase shift, we computed the probability of observing the phase shift regime in T+h th week, given the data of the values of the largest share among the sub-lineage groups up to T th week. The detailed estimation of the probability of the phase shift state and forecasting probability of the phase shift is shown in Supplementary Material S4.

All statistical analyses for our suggested model were performed using GAUSS Software (version 16, USA), and the explanatory data analyses were conducted using EViews (version 8, USA).

### Explanatory data analysis

This section presents the results of the explanatory data analysis for the max share variable $y_{t}$ . Specifically, we tested if the max share variable in each county has a unit root and if the error term in the ARMA model ( $\epsilon_{t}$ in equation 1) follows a normal distribution. If the null hypothesis of the normal distribution is rejected, $\epsilon_{t}$ would have clustered volatility, which implies heteroskedasticity of the error term and supports our motivation for the time-varying volatility model.

We found that the existence of a unit root cannot be rejected using the standard Dicky-Fuller unit root test. As shown in Supplementary Material S5, the *P*-values of the test statistics were larger than 10% for all cases. We also confirm that the estimation result of the regime probability is very similar to the first-differenced series of the max share variable for each country.

For the normality test of the error terms, we conducted Jarque-Bera normal distribution test (16). We found that the null hypothesis of the normal distribution was strongly rejected in each case, except in the case of Korea (Supplementary Material S6). Because we did not find strong evidence of volatility clustering for the error term of the Korean max share, we tested the heteroskedasticity of $\epsilon_{t}$ in the Korean case by checking if the mean of square error term, which is equal to Var ( $\epsilon_{t}$ ), is time varying. As presented in Supplementary Material S6, the Quandt-Andrews unknown breakpoint test (17, 18) statistics (maximum likelihood ratio F-statistics) strongly rejected the null hypothesis that the mean of $\epsilon_{t}^{2}$ is time constant in our sample period. The test results imply that the volatilities of the max share variables in all cases in our study have time-varying volatility. The Quandt-Andrews unknown breakpoint test is used to check if a model parameter in a time-series model has at least one structural break without assuming break periods. In our test, we used the extended data from Korea to match the starting period of the test sample (5% trimmed data) with our model estimation period.

## RESULTS

**Fig 1:**
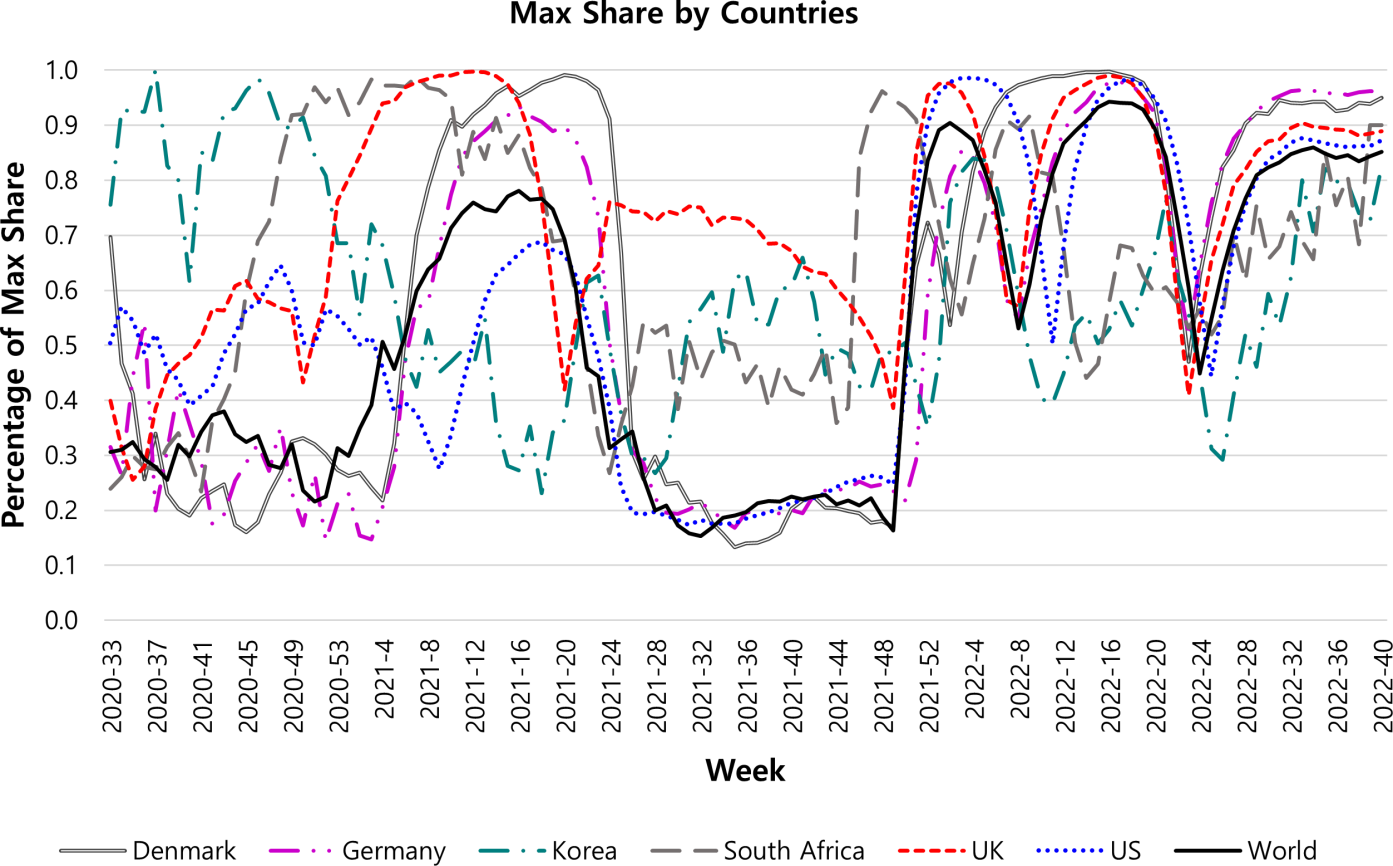
Result of the Markov-switching model for the max share of sub-lineages, including Denmark, Germany, Korea, South Africa, the United Kingdom, the United States, and worldwide. The *Y*-axis is the percentage of the max share, and the *X*-axis is the weekly time.

Figure 1 shows the max share of the sub-lineage, including Denmark, Germany, Korea, South Africa, the United Kingdom, the United States, and worldwide during the observable period. The max shares of all countries were approximately 20 to 100 percentage points, Korea’s max share was over 80%, and the dominant sub-lineage was B.1.497, which reflected the success of Korea’s quarantine policy regarding the sample size in 2020. In Denmark, B.1.1, B.1.160, and B.1.177.21 had the largest shares in 2020; B.1.1 and B.1.351 (Beta) in South Africa; B.1.1 and B.1.177 in the UK; and B.1 and B.1.2 in the US. In Germany, B.1.1, AY.43, B.1.177, B.1.258, and B.1.221 shared dominance. These different dominances by countries are observed at the max share, which was about 20 to 30 percentage points. In particular, the AY.43 (Delta, B.1.617.2.43) variant is observed as a dominant variant in the 37th week of 2020 in Germany. The AY.43 variant in Germany is important because it appeared again in the middle of 2021. In summary, SARS-CoV-2 variants competed for dominance in this period in each country and worldwide.

After this phase, the B.1.1.7 (Alpha) variant arose in the UK in the 51st week of 2020. The Alpha led the trend of sub-lineage dominance, spreading to the other two countries in Europe and the United States. However, the Beta variant still dominated in South Africa, and A.18, B.1.497, and B.1.619.1 shared their dominance except for 3 weeks in Korea. In the countries where Alpha variant dominates, its max shares over countries peaked at about 70 to over 90 percentage points. This phase is led by the Alpha variant.

From the 20th to the 30th week in 2021, the variants of B.1.617.2 (Delta, AY lineage) rapidly dominated instead of Alpha in the four countries of Europe and the United States, B.1.619.1 in Korea, and Beta in South Africa. Despite AY.4 dominating Worldwide, each max share of countries except the United Kingdom was dominated by different variants of Delta. For example, AY.7.1, AY.43, and AY.122 shared the dominance with AY.4 in Denmark, AY.69 mainly in Korea, AY.45 mainly in South Africa, and AY.44 and AY.103 shared in the United States. In particular, AY.43, which dominated once in the 37th week in 2020 in Germany, re-dominated again in the 29th week and 36th to 50th week in 2021. The changes in the dominant variance in all regions revealed that another competition appeared among Delta variants in this period. Specifically, the max share values of open and vast countries in their continents were under 50 percentage points.

After this “Delta phase,” the Omicron variant spreads far more rapidly than the Delta variant from South Africa to the rest of the world. The unprecedented spread and dominance of Omicron BA.1* are observed in Fig. 1 from the 46th week of 2021. BA.2* and BA.5* consecutively followed the dominance of BA.1* except for South Africa. In South Africa, BA.4* succeeded the dominance of BA. 2* and was succeeded in its dominance by BA.5*. These dominance changes across countries mostly synchronized all over the world in 2022. Readers can refer to Supplementary Material S3 for the dominance changes described above.

**Fig 2:**
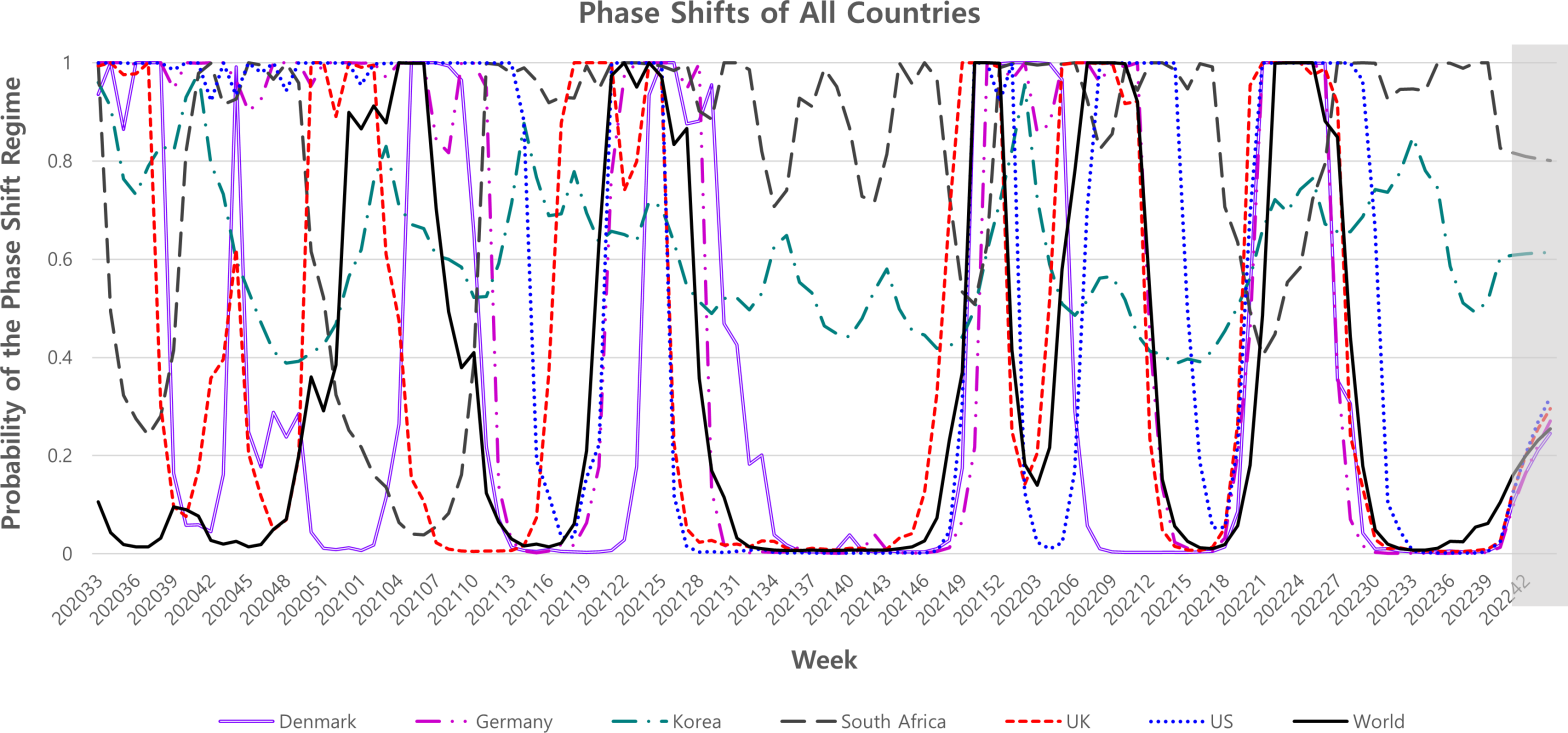
Result of the Markov-switching model for the inferred probability of the phase shift (high volatility) regime, including Denmark, Germany, Korea, South Africa, the United Kingdom, the United States, and worldwide. The *Y*-axis is the probability of the phase shift, and the *X*-axis is the weekly time.

**Fig 3:**
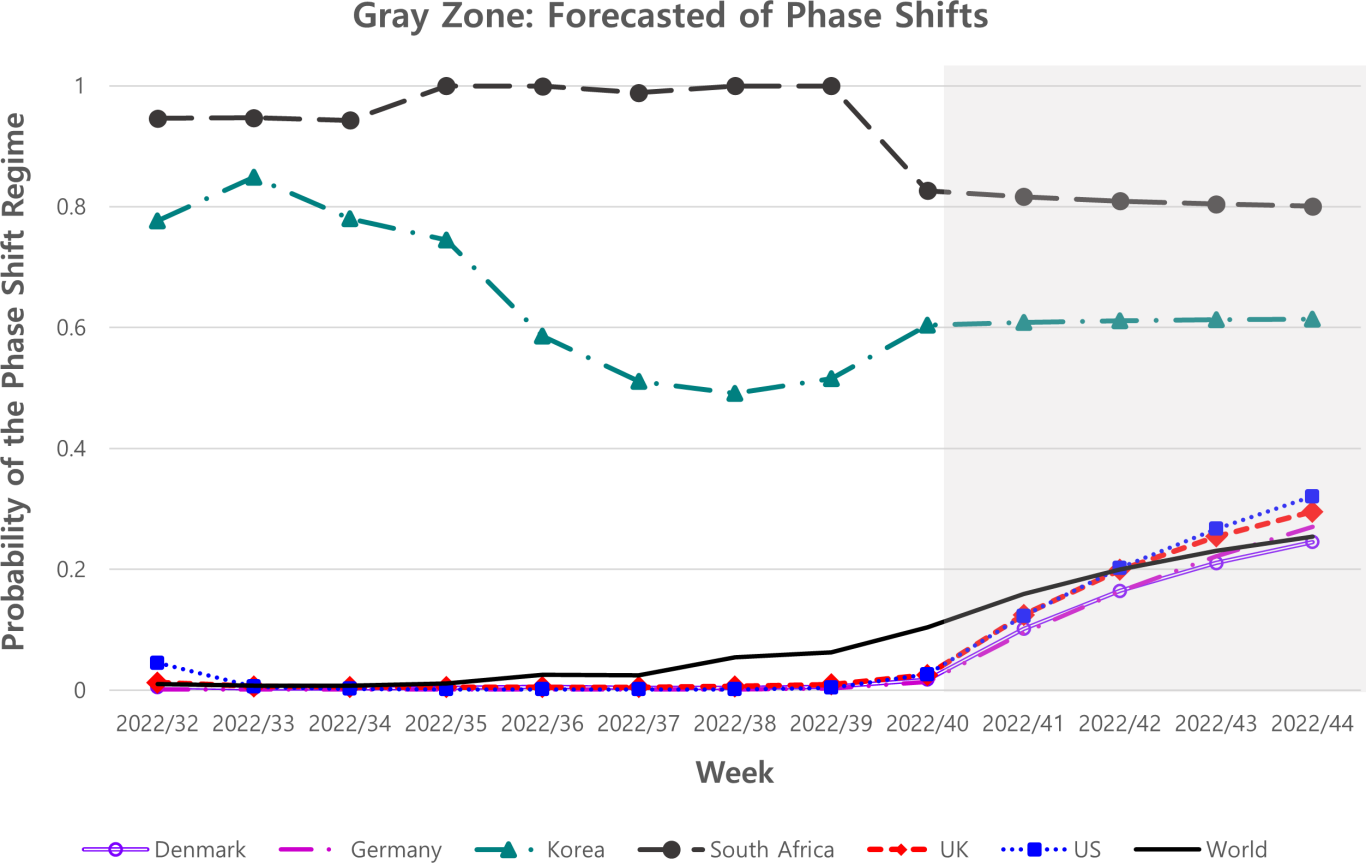
A zoom-in graph of Fig. 2 focuses on the phase shift regime for prediction, including Denmark, Germany, Korea, South Africa, the United Kingdom, the United States, and Worldwide. The *Y*-axis is the probability of the phase shift, and the *X*-axis is the weekly time. The darker the color is the forecasting of the phase shift regime.

Figure 2 shows the inferred probability of the phase shift regime and how the probability oscillates between two volatility statuses—one (high) and zero (low) in all regions. As the inferred probability is close to 1, the corresponding week is more likely to be in the high-volatility status. As presented in Fig. 2, the high-volatility regime is well corresponded to the periods with the rank change or rank competence, and the low-volatility regime corresponds to the weeks with stable values of the max share. Supplementary Material S7 shows separated figures depending on countries for the max share and the probability of phase shift. Figure 3 shows a zoom-in graph of Fig. 2 focusing on the weeks of the phase shift regime for prediction. After the 40th week of 2022, the probability of observing the phase shift regime stays below 50% in 4 weeks, indicating low likelihood of rank change in one month after the end period of our samples.

Table 1 presents the estimated parameters containing the value of the inferred transition probabilities $p_{00}$ and $p_{11}$ defined in the two-state Markov-switching process, indicating the probability of staying on the same regime as the previous week. In other words, $p_{00}$ ( $p_{11})$ ) is the probability that a stable dominance (phase shift) regime persists in the next period. In Table 1, $p_{00}$ and $p_{11}$ of worldwide show significantly high probabilities of 92.02% and 83.55%, respectively. These two parameters can be interpreted as the transition probabilities that next week’s regime stays unchanged in the current week. In other words, the estimated probabilities indicate that the stable dominance and phase shift regime will continue in the next week with a chance of 92.02% and 83.55%, respectively. $p_{00}$ ranges from 76% to 92%, and $p_{11}$ ranges from 83% to 94%, depending on the country. As shown in Table 1, the estimated value of conditional variances in the phase-shift regime ( $\sigma_{high}^{2}$ ) is significantly higher than in the stable dominance regime ( $\sigma_{low}^{2}$ ). In the table, the values of the AR[1] coefficients are close to 1, implying a unit root in each max share series. Since the max share series follows *I*[1] process, we presented the random walk R-squared as a measure of the fit of the ARMA model instead of the conventional R-squared that asymptotically converges 1 if a unit root exists. Random walk R-squared uses the pure random walk as the benchmark to evaluate the model fitness. As the ARMA model has a better fit compared to the random walk, the random walk R-squared becomes closer to 100%.

**TABLE 1:** Model parameter estimations from the two-state Markov-switching process^*a*^

	Denmark			Germany			Korea			South Africa			United Kingdom			United States			Worldwide		
	Coeff.	S.E.	*t*-Value	Coeff.	S.E.	*t*-Value	Coeff.	S.E.	*t*-Value	Coeff.	S.E.	*t*-Value	Coeff.	S.E.	*t*-Value	Coeff.	S.E.	*t*-Value	Coeff.	S.E.	*t*-Value
Mu	0	0.01	0.3	0	0.01	0.42	0.08	0.03	2.51	0.03	0.02	1.49	0.01	0.03	0.3	0	0	0.99	0	0.01	0.37
Phi	1	0.01	121.93	1	0.01	123.33	0.86	0.05	16.4	0.96	0.02	39.07	0.99	0.03	34.59	1	0.01	172.26	0.99	0.01	77.11
Delta	0.26	0.06	4.55	0.42	0.07	6.17	0.13	0.09	1.41	0.02	0.1	0.23	0.47	0.06	7.32	0.46	0.04	10.48	0.38	0.08	5.01
Sig2_0low	0	0	3.54	0	0	4.43	0.005	0.006	0.89	0.001	0.001	1.81	0	0	2.66	0	0	3.82	0.001	0	4.07
Sig2_1high	0.016	0.004	3.76	0.012	0.002	5.31	0.01	0.006	1.58	0.009	0.002	5.42	0.009	0.002	4.18	0.006	0.001	5.52	0.007	0.002	3.64
*p*00	0.91	0.04	24.38	0.92	0.04	21.19	0.76	0.43	1.77	0.77	0.13	5.75	0.89	0.05	18.86	0.9	0.05	19.76	0.92	0.04	24.31
*p*11	0.83	0.07	11.94	0.93	0.04	23.27	0.85	0.58	1.46	0.94	0.06	16.38	0.85	0.06	13.78	0.92	0.04	25.96	0.84	0.07	11.17
Log-likelihood	197.38			178.22			112.28			121.71			198.17			215.26			201.65		
Random walk *R*^2^ (%)	17			11.61			7.7			4.1			26.13			28.84			24.05		

^*a*^ Coeff, coefficient, S.E., standard error.

## DISCUSSION

This study examined the changes in different weekly patterns of SARS-CoV-2 based on dominant variants in six countries and worldwide using the max share of the sub-lineage. The estimated probabilities indicate that the stable dominance and phase shift regime will continue in the next week on the two-state Markov-switching process. The large estimated values of $p_{00}$ and $p_{11}$ in Table 1 indicate that the stable dominance and phase shift regimes are highly persistent in the six countries and worldwide. The persistent stable dominance regimes indicate that once a variant becomes dominant, it is highly possible to continue having the largest share among the variants in the next several weeks. Likewise, the highly persistent phase shift regimes imply that once strong competition among the SARs-CoV-2 variants happens, it is highly likely to last multiple weeks.

Figure 2 presents that time lags exist among the inferred probabilities of the phase shift regime corresponding to some SARs-CoV-2 variants in different countries. Due to each government utilizing differing COVID-19 policies, some countries had different outbreaks of SARS-CoV-2 variants. For example, the Delta variant dominated in the United Kingdom from the 20th week of 2021, and the same variant dominated in South Africa from the 24th week of 2021. However, Korea discovered the Delta variant in the 27th week of 2021. As a result, there were 4-week lags of the dominant Delta variant between the United Kingdom and South Africa. Simultaneously, there was a 3-week time lag between the dominant Delta variant between South Africa and Korea. A recent comparative study of the time lag effects of COVID-19 policies has shown that different policies, such as travel bans with quarantine, may have different effects on the COVID-19 pandemic (19, 20). However, even with each government utilizing different COVID-19 policies, we can predict the outbreak of SARS-CoV-2 variants using the inferred probabilities of the phase shift regime in this study.

According to Fig. 1 and 2, the changes in max share and phase shift regime indicate the spread of new emerging strains of coronavirus mutations. In other words, these figures show the appearance of many SARS-CoV-2 variants due to the different levels of genetic changes (21). The phase shift also shows different patterns depending on countries. For example, since Denmark, Germany, the United States, and the United Kingdom were dominated by the Alpha variant, the probability of the phase shift was close to 0 from the 51st week of 2020 to the 26th week of 2021. In contrast, the Beta variant decreased the probability of phase shift in South Africa. During the same period, Korea continuously kept a strict quarantine policy so the probability of phase shift is close to 1. When the epidemic lineage of coronavirus was classified by the outbreak of the mutant virus, the quarantine policies implemented in each country were different. During the Alpha and the Delta era, each country implemented strict quarantine policies, such as travel restrictions or lockdowns, to adequately control coronavirus transmission between countries (22). Whereas, in the era of the Omicron variant, coronavirus transmission between countries spreads quickly despite highly accurate polymerase chain reaction tests and vaccination (23, 24). In addition, the Delta variant was more pathogenic and deadly to humans, while the Omicron variant was more infectious and immune escaping (25, 26). For this reason, this study considers the Delta and Omicron variants to have the characteristics of mutant viruses and were, therefore, used for predicting mutant viruses.

In Fig. 1, the probability of the phase shift regime in Korea and South Africa had a different pattern than in European countries and the United States. Notably, in the Omicron era, Korea has continuously implemented policies, such as social distancing, strict border management, vaccination, and wearing a mask, in contrast to other countries due to local community transmission (27). Since strict COVID-19 policies are evident in Korea, the probability of the phase shift moved between 0.4 and 0.8, which means the volatility of dominant variant was low. In South Africa, the Omicron variant was first identified on 25 November 2021 (28) and mutated to sub-variant BA.4 and BA.5 in 2022. The Omicron variant evolved when vaccination coverage was 36.0% among 12 years or older, with only 20.1% taking at least two doses of the coronavirus vaccine (29). Such variant volatility as an infection wave is associated with increased transmissibility, patient disease severity, and reduced neutralization by antibodies from vaccination. In addition, the existing vaccines could be ineffective against new variants because other mutations could not be involved in immune escape (30). Thus, we can infer the emerging variant from high-volatility regime through the phase shift regime.

Figure 3 describes the 4 weeks of forecasting variant volatility after the 40th week of 2022. Korea and South Africa were dominated by the current regime tendencies, while worldwide and other countries competed for a probability between the current regime and a new variant. Prior studies used several statistical methods to forecast the number of patients, recoveries, or deaths in the COVID-19 pandemic, such as the modified SIR model (6) and the ARIMA model (7). However, the accuracy of their COVID-19 predictions remained questionable, despite using various existing forecasting models. Such traditional analysis models do not immediately include dynamic human behaviors, changing public health policies, and many other ambiguous factors (7). However, in this study, the Markov-switching model can easily predict the subsequent waves using the probability of observing the phase shift regime as volatility without other variables. In other words, our approach provides information about whether a current dominant variant will continue to have the largest share or a new one will emerge as a candidate for the new dominant variant. In addition to the Markov-switching model based on the maximum likelihood estimation and a linear system, this model is a valuable tool for forecasting the approach of variants (31).

Our study has several strengths. First, we used the sub-lineages in six countries from the PANGO database. Using the sub-lineages, we could monitor the recombinant sequences of SARS-CoV-2 mutations (posted by De Majo N, https://virological.org/t/issues-with-sars-cov-2-sequencing-data/473) (32). Second, we applied the Markov-switching model to predict emerging variants as the next wave using regime volatility. Most previous studies were designed to predict COVID-19 using time series or epidemical models. However, these models were not sufficient to consider other ambiguous factors and did not immediately include the current information for forecasting COVID-19.

Our study had limitations, in that the Markov-switching model in this study assumed the probability of facing the phase shift regime, given the information about past states to be time constant. Suppose a significant factor explaining the growth in the case number of each new variant becomes available. In this case, we can extend the model by allowing time-varying transition probabilities depending on such a factor. The model extension would enhance the accuracy of describing past observations and forecasting future regimes. Since our goal is to identify the state of the ranks among the sub-lineages, the model in this study estimates the time-varying conditional variance assuming discrete regime changes. For this reason, the estimated and forecasted values of the conditional variance of the max share would be less accurate than models using continuous time-varying volatility specifications. If the research purpose is more focused on forecasting the value of the volatility instead of the regime, using continuous-type models might be recommended. Furthermore, models using Markov transitions also have inherent limitations. In particular, the time series model may not adequately capture the effects of various intervention policies and the reduction of variation as the COVID-19 epidemic transitions toward endemicity. To better understand the likelihood of new mutant virus emergence over time, our model requires repeated measurements over an extended period during the epidemic.

In conclusion, although our study has acknowledged limitations, we have successfully developed a model that leverages global virus surveillance data to generate approximate time-series predictions of new variant emergence frequency in each country. This model is expected to contribute to a more comprehensive understanding of the pandemic’s progression and response strategies. By aiding in determining the appropriate update frequency of vaccines or the duration of non-pharmacological interventions, our approach offers valuable insights for addressing the ongoing challenges posed by COVID-19.

## Data Availability

The data sets generated on March 2023 from covSPECTRUM (https://cov-spectrum.org/explore/World/AllSamples/AllTimes/variants), and/or analyzed during the current study are available from a Github repository of Gil-Artificial Intelligence and Big-Data Convergence (G-ABC) Center (https://github.com/G-ABC-Infection/AI-Infection/tree/main/Data/Phase%20Shift%20Data_original).

## SUPPLEMENTAL MATERIAL

Supplementary materials S1-7 spectrum.01669-23-s0001.pdf Supplementary tables, figures and methods.

ASM does not own the copyrights to Supplemental Material that may be linked to, or accessed through, an article. The authors have granted ASM a non-exclusive, world-wide license to publish the Supplemental Material files. Please contact the corresponding author directly for reuse.

## References

[1] Dawood AA . 2020. Mutated COVID**-19** may foretell a great risk for mankind in the future. New Microbes New Infect 35:100673. doi:10.1016/j.nmni.2020.100673 32292587 PMC7129032

[2] Tang X , Wu C , Li X , Song Y , Yao X , Wu X , Duan Y , Zhang H , Wang Y , Qian Z , Cui J , Lu J . 2020. On the origin and continuing evolution of SARS-CoV-2. Natl Sci Rev 7:1012–1023. doi:10.1093/nsr/nwaa036 34676127 PMC7107875

[3] Wassenaar TM , Wanchai V , Buzard G , Ussery DW . 2022. The first three waves of the COVID-19 pandemic hint at a limited genetic repertoire for SARS-CoV-2. FEMS Microbiol Rev 46:fuac003. doi:10.1093/femsre/fuac003 35076068 PMC9075578

[4] Vasireddy D , Vanaparthy R , Mohan G , Malayala SV , Atluri P . 2021. Review of COVID-19 variants and COVID-19 vaccine efficacy: what the clinician should know? J Clin Med Res 13:317–325. doi:10.14740/jocmr4518 34267839 PMC8256910

[5] Luo J . 2021. Forecasting COVID-19 pandemic: unknown unknowns and predictive monitoring. Technol Forecast Soc Change 166:120602. doi:10.1016/j.techfore.2021.120602 33495665 PMC7817405

[6] Cooper I , Mondal A , Antonopoulos CG . 2020. A SIR model assumption for the spread of COVID-19 in different communities. Chaos Solitons Fractals 139:110057. doi:10.1016/j.chaos.2020.110057 32834610 PMC7321055

[7] Aslam M . 2020. Using the kalman filter with Arima for the COVID-19 pandemic dataset of Pakistan. Data Brief 31:105854. doi:10.1016/j.dib.2020.105854 32572378 PMC7292003

[8] Wang P , Zheng X , Ai G , Liu D , Zhu B . 2020. Time series prediction for the epidemic trends of COVID-19 using the improved LSTM deep learning method: case studies in Russia, Peru and Iran. Chaos Solitons Fractals 140:110214. doi:10.1016/j.chaos.2020.110214 32839643 PMC7437443

[9] Shiferaw YA . 2021. Regime shifts in the COVID-19 case fatality rate dynamics: a Markov-switching autoregressive model analysis. Chaos Solitons Fractals 6:100059. doi:10.1016/j.csfx.2021.100059

[10] de Oliveira AMB , Binner JM , Mandal A , Kelly L , Power GJ . 2021. Using GAM functions and Markov-switching models in an evaluation framework to assess countries' performance in controlling the COVID-19 pandemic. BMC Public Health 21:2173. doi:10.1186/s12889-021-11891-6 34837982 PMC8626735

[11] Psaradakis Z , Spagnolo N . 2003. On the determination of the number of regimes in Markov-switching autoregressive models. J Time Ser Anal 24:237–252. doi:10.1111/1467-9892.00305

[12] Ma R , Zheng X , Wang P , Liu H , Zhang C . 2021. The prediction and analysis of COVID-19 epidemic trend by combining LSTM and Markov method. Sci Rep 11:17421. doi:10.1038/s41598-021-97037-5 34465820 PMC8408143

[13] GISAID . 2023. CoV-spectrum. Available from: https://cov-spectrum.org/explore/World/AllSamples/AllTimes/variants

[14] Hamilton JD . 1989. A new approach to the economic-analysis of nonstationary time-series and the business-cycle. Econometrica 57:357. doi:10.2307/1912559

[15] Kim CJ . 1994. Dynamic linear-models with Markov-switching. J Econom 60:1–22. doi:10.1016/0304-4076(94)90036-1

[16] Jarque CM , Bera AK . 1987. A test for normality of observations and regression residuals. Int Stat Rev 55:163. doi:10.2307/1403192

[17] Andrews DWK . 1993. Tests for parameter instability and structural-change with unknown change-point. Econometrica 61:821. doi:10.2307/2951764

[18] Quandt RE . 1960. Tests of the hypothesis that a linear regression system obeys two separate regimes. JASA 55:324–330. doi:10.1080/01621459.1960.10482067

[19] Bian Z , Zuo F , Gao J , Chen Y , Pavuluri Venkata SSC , Duran Bernardes S , Ozbay K , Ban XJ , Wang J . 2021. Time lag effects of COVID-19 policies on transportation systems: a comparative study of New York city and Seattle. Transp Res Part A Policy Pract 145:269–283. doi:10.1016/j.tra.2021.01.019 36569966 PMC9759401

[20] Stübinger J , Schneider L . 2020. Epidemiology of coronavirus COVID-19: forecasting the future incidence in different countries. Healthcare (Basel) 8:99. doi:10.3390/healthcare8020099 32326512 PMC7349853

[21] Jia Z , Gong W . 2021. Will mutations in the spike protein of SARS-CoV-2 lead to the failure of COVID-19 vaccines? J Korean Med Sci 36:e124. doi:10.3346/jkms.2021.36.e124 33975397 PMC8111046

[22] Khatib AN , McGuinness S , Wilder-Smith A . 2021. COVID-19 transmission and the safety of air travel during the pandemic: a scoping review. Curr Opin Infect Dis 34:415–422. doi:10.1097/QCO.0000000000000771 34524196

[23] Yuan P , Aruffo E , Tan Y , Yang L , Ogden NH , Fazil A , Zhu H . 2022. Projections of the transmission of the Omicron variant for Toronto, Ontario, and Canada using surveillance data following recent changes in testing policies. Infect Dis Model 7:83–93. doi:10.1016/j.idm.2022.03.004 35372735 PMC8964508

[24] Qian Y , Cao S , Zhao L , Yan Y , Huang J . 2022. Policy choices for Shanghai responding to challenges of Omicron. Front Public Health 10:927387. doi:10.3389/fpubh.2022.927387 36016887 PMC9395601

[25] Wang L , Zhou HY , Li JY , Cheng YX , Zhang S , Aliyari S , Wu A , Cheng G . 2022. Potential intervariant and intravariant recombination of Delta and Omicron variants. J Med Virol 94:4830–4838. doi:10.1002/jmv.27939 35705528 PMC9350351

[26] Kannan SR , Spratt AN , Sharma K , Chand HS , Byrareddy SN , Singh K . 2022. Omicron SARS-CoV-2 variant: unique features and their impact on pre-existing antibodies. J Autoimmun 126:102779. doi:10.1016/j.jaut.2021.102779 34915422 PMC8666303

[27] Lee JJ , Choe YJ , Jeong H , Kim M , Kim S , Yoo H , Park K , Kim C , Choi S , Sim J , Park Y , Huh IS , Hong G , Kim MY , Song JS , Lee J , Kim EJ , Rhee JE , Kim IH , Gwack J , Kim J , Jeon JH , Lee WG , Jeong S , Kim J , Bae B , Kim JE , Kim H , Lee HY , Lee SE , Kim JM , Park H , Yu M , Choi J , Kim J , Lee H , Jang EJ , Lim D , Lee S , Park YJ . 2021. Importation and transmission of SARS-CoV-2 B.1.1.529 (Omicron) variant of concern in Korea, November 2021. J Korean Med Sci 36:e346. doi:10.3346/jkms.2021.36.e346 34962117 PMC8728587

[28] Callaway E . 2021. Heavily mutated Omicron variant puts scientists on alert. Nature 600:21–21. doi:10.1038/d41586-021-03552-w 34824381

[29] Madhi SA , Kwatra G , Myers JE , Jassat W , Dhar N , Mukendi CK , Nana AJ , Blumberg L , Welch R , Ngorima-Mabhena N , Mutevedzi PC . 2022. Population immunity and COVID-19 severity with Omicron variant in South Africa. N Engl J Med 386:1314–1326. doi:10.1056/NEJMoa2119658 35196424 PMC8908853

[30] Gao S-J , Guo H , Luo G . 2022. Omicron variant (B.1.1.529) of SARS-CoV-2, a global urgent public health alert! J Med Virol 94:1255–1256. doi:10.1002/jmv.27491 34850421 PMC9015397

[31] Arumugam R , Rajathi M . 2020. Markov model for prediction of corona virus COVID-19 in India- a statistical study. JXU 14:1422–1426. doi:10.37896/jxu14.4/164

[32] De Majo N , Walker C , Borges R , Weilguny L , Slodkowicz G , Goldman N . 2020. Issues with SARS-CoV-2 sequencing data.

