# Supplementary material for: Inference and forecasting phase shift regime of COVID-19 sub-lineages with a Markov-switching model
Source: Microbiol Spectr. 2023 Oct 9;11(6):e01669-23. doi: 10.1128/spectrum.01669-23 (PMC10714866; doi:10.1128/spectrum.01669-23)
Supplement: Supplementary materials S1-7 — Supplementary tables, figures and methods. [file spectrum.01669-23-s0001.pdf]

Supplementary materials

Inference and Forecasting Phase Shift Regime of COVID-19 Sub-lineages with a Markov Switching Model

Supplementary Materials S1. Comparison of the fitness measures with GARCH model

	RMSE		MAE		MAPE	
	GARCH	MS	GARCH	MS	GARCH	MS
Denmark	0.078	0.074	0.048	0.042	0.133	0.103
Germany	0.081	0.080	0.050	0.047	0.142	0.140
Korea	0.090	0.090	0.071	0.072	0.135	0.135
South Africa	0.086	0.085	0.064	0.063	0.117	0.113
United Kingdom	0.067	0.061	0.041	0.038	0.068	0.064
United States	0.060	0.058	0.038	0.036	0.076	0.071
World	0.053	0.053	0.038	0.034	0.115	0.094

Note: GARCH model refers to GARCH (1,1) specification, and MS refers to the Markov-switching volatility model. RMSE, MAE, and MAPE refers to root mean squared error, mean absolute error, and mean absolute percentage error.

Supplementary materials S2. Sub-lineage Data Reference

WHO label	PANGO	Lineage	Denmark Sub-lineage	Germany Sub-lineage	Korea Sub-lineage	South Africa Sub-lineage	United Kingdom Sub-lineage	United States Sub-lineage	World Sub-lineage
	A				A, A.18				
	B				B		AD.2, B	B	B, D.2
	B.1		B.1, B.1.1, B.1.1.70, B.1.1.277 B.1.1.298, B.1.1.433, B.1.36 B.1.160, B.1.221, B.1.258 B.1.258.11, B.1.525	B.1, B.1.1, B.1.1.317, B.1.160, B.1.221, B.1.258, B.1.525	B.1, B.1.1, B.1.429 B.1.466.2, B.1.470, B.1.497, B.1.619, B.1.619.1, B.1.620	B.1, B.1.1, B.1.1.1, B.1.1.34, B.1.1.52, B.1.1.53, B.1.1.54, B.1.1.57, B.1.1.117, B.1.1.273, B.1.1.412, B.1.1.459, B.1.1.507, B.1.1.528, B.1.237 B.1.381, B.1.617	B.1, B.1.1, B.1.1.37 B.1.36, B.1.160, B.1.258	B.1, B.1.1, B.1.1.519, B.1.2 B.1.234, B.1.240, B.1.243 B.1.427, B.1.429, B.1.526 B.1.621, B.1.637	B.1, B.1.1, B.1.1.214, B.1.1.519, B.1.2 B.1.160, B.1.221, B.1.243, B.1.258, B.1.427, B.1.429, B.1.526, B.1.621, B.1.637
	B.1.1.1	C				C.1, C.1.2			
Alpha	B.1.1.7	Q	B.1.1.7, Q.7	B.1.1.7, Q.7	B.1.1.7	B.1.1.7	B.1.1.7	B.1.1.7, Q.3	B.1.1.7
Beta	B.1.351			B.1.351	B.1.351	B.1.351			B.1.351
Gamma	P.1				P.1			P.1, P.1.17	P.1, P.1.14
Delta	B.1.617.2	AY	B.1.617.2, AY.4, AY.4.2 AY.4.5, AY.4.6, AY.4.10 AY.5, AY.7.1, AY.9.2,AY.33, AY.36, AY.42, AY.43, AY.43.6, AY.46, AY.46.6 AY.80, AY.98.1, AY.111 AY.120.2.1, AY.121, AY.122 AY.122.3, AY.125,	B.1.617.2, AY.4, AY.4.2, AY.4.2.3 AY.4.4, AY.5 AY.7.1, AY.9.2 AY.33, AY.34, AY.36, AY.41 AY.42, AY.43 AY.46, AY.46.6 AY.84, AY.98.1 AY.112, AY.121 AY.121.1, AY.122 AY.125, AY.126 AY.127, AY.129	B.1.617.2, AY.4, AY.23, AY.24, AY.33, AY.43, AY.44, AY.69, AY.75, AY.78, AY.103, AY.121, AY.122, AY.126	B.1.617.2, AY.6, AY.19, AY.32, AY.38 AY.45, AY.46, AY.91 AY.99, AY.107, AY.116, AY.120 AY.120.2, AY.122	B.1.617.2, AY.4 AY.4.2, AY.4.2.1 AY.4.2.2, AY.4.8 AY.43, AY.46.5 AY.5, AY.6, AY.7, AY.9 AY.9.2, AY.36 AY.98, AY.98.1 AY.111, AY.120 AY.122, AY.127	B.1.617.2, AY.3 AY.3.1, AY.4, AY.13 AY.14, AY.20, AY.25 AY.25.1, AY.25.1.1 AY.26, AY.39, AY.39.1,AY.43 AY.44, AY.46.4, AY.47, AY.54, AY.75 AY.100, AY.103 AY.113, AY.114 AY.116.1, AY.117 AY.118, AY.119 AY.119.2, AY.120.1 AY.122	B.1.617.2,AY.3, AY.3.1, AY.4 AY.4.2, AY.4.2.1, AY.4.2.2 AY.4.5, AY.4.6, AY.5, AY.6 AY.9, AY.9.2, AY.14, AY.20 AY.23, AY.25, AY.25.1, AY.26 AY.27, AY.29, AY.33, AY.36 AY.39, AY.39.1, AY.42, AY.43 AY.44, AY.46, AY.46.5, AY.46.6, AY.47, AY.54, AY.75 AY.98, AY.98.1, AY.99.2 AY.100, AY.103, AY.117 AY.118,AY.119, AY.120

			AY.126<br>AY.127, AY.129						AY.121, AY.122, AY.125<br>AY.126, AY.127, AY.129
	B.1.177		B.1.177, B.1.177.12,<br>B.1.177.21, B.1.177.35<br>B.1.177.60, B.1.177.86	B.1.177, B.1.177.81<br>B.1.177.86	B.1.177		B.1.177, B.1.177.4,<br>B.1.177.7<br>B.1.177.57		B.1.177
Omicron	B.1.1.529	BA	B.1.1.529	B.1.1.529	B.1.1.529	B.1.1.529	B.1.1.529	B.1.1.529	B.1.1.529
		BA.1	BA.1, BA.1.1. BA.1.1.1<br>BA.1.1.2. BA.1.1.4.<br>BA.1.1.7<br>BA.1.1.10. BA.1.1.11,<br>BA.1.1.13, BA.1.1.14,<br>BA.1.1.15, BA.1.1.16,<br>BA.1.1.18, BA.1.3,<br>BA.1.6<br>BA.1.7, BA.1.8,<br>BA.1.9. BA.1.10,<br>BA.1.12, BA.1.13<br>BA.1.13.1, BA.1.14,<br>BA.1.14.1<br>BA.1.14.2, BA.1.15,<br>BA.1.15.1<br>BA.1.15.2, BA.1.15.3,<br>BA.1.16<br>BA.1.16.1, BA.1.16.2,<br>BA.1.17<br>BA.1.17.2, BA.1.18,<br>BA.1.19<br>BA.1.20, BA.1.21.1,<br>BA.1.22<br>BA.1.24, BD.1	BA.1, BA.1.1,<br>BA.1.1.1, BA.1.1.2<br>BA.1.1.3, BA.1.1.4<br>BA.1.1.5, BA.1.1.7<br>BA.1.1.8, BA.1.1.9<br>BA.1.1.10, BA.1.1.11<br>BA.1.1.12, BA.1.1.13<br>BA.1.1.14, BA.1.1.15<br>BA.1.1.16, BA.1.1.17<br>BA.1.1.18, BA.1.3<br>BA.1.5, BA.1.6,<br>BA.1.7, BA.1.8,<br>BA.1.9, BA.1.10,<br>BA.1.12, BA.1.13<br>BA.1.13.1, BA.1.14<br>BA.1.14.1, BA.1.14.2<br>BA.1.15, BA.1.15.1<br>BA.1.15.2, BA.1.15.3<br>BA.1.16, BA.1.16.2<br>BA.1.17, BA.1.17.1<br>BA.1.17.2, BA.1.18<br>BA.1.19, BA.1.20<br>BA.1.21.1, BA.1.22<br>BA.1.23, BA.1.24<br>BC.2, BD.1	BA.1, BA.1.1,<br>BA.1.1.1, BA.1.1.2,<br>BA.1.1.4, BA.1.1.5,<br>BA.1.1.10,BA.1.1.11<br>BA.1.1.14, BA.1.1.15,<br>BA.1.1.16, BA.1.1.18,<br>BA.1.3, BA.1.6,<br>BA.1.7, BA.1.9,<br>BA.1.10, BA.1.12,<br>BA.1.13, BA.1.13.1,<br>BA.1.14, BA.1.14.1,<br>BA.1.15, BA.1.15.1,<br>BA.1.15.3, BA.1.16,<br>BA.1.16.1, BA.1.17,<br>BA.1.17.2, BA.1.18,<br>BA.1.19, BA.1.20,<br>BA.1.22, BD.1	BA.1, BA.1.1.<br>BA.1.1.1, BA.1.3<br>BA.1.6. BA.1.9,<br>BA.1.10, BA.1.12<br>BA.1.13, BA.1.14<br>BA.1.15, BA.1.17<br>BA.1.17.2, BA.1.18<br>BA.1.19, BA.1.21.1<br>BA.1.23, BA.1.24	BA.1, BA.1.1, BA.1.1.1,<br>BA.1.1.2, BA.1.1.3,<br>BA.1.1.4, BA.1.1.5,<br>BA.1.1.6, BA.1.1.7,<br>BA.1.1.8, BA.1.1.9,<br>BA.1.1.10, BA.1.1.11,<br>BA.1.1.12, BA.1.1.13,<br>BA.1.1.14, BA.1.1.15,<br>BA.1.1.16, BA.1.1.17,<br>BA.1.1.18, BA.1.2,<br>BA.1.3<br>BA.1.5, BA.1.6<br>BA.1.7, BA.1.8<br>BA.1.9, BA.1.10<br>BA.1.12, BA.1.13<br>BA.1.13.1, BA.1.14,<br>BA.1.14.1, BA.1.14.2,<br>BA.1.15, BA.1.15.1,<br>BA.1.15.2, BA.1.15.3,<br>BA.1.16, BA.1.16.1,<br>BA.1.16.2, BA.1.17,<br>BA.1.17.1, BA.1.17.2,<br>BA.1.18, BA.1.19<br>BA.1.20, BA.1.21.1,<br>BA.1.22, BA.1.24<br>BC.2, BD.1	BA.1, BA.1.1, BA.1.1.1,<br>BA.1.1.2<br>BA.1.1.3, BA.1.1.4<br>BA.1.1.5, BA.1.1.6<br>BA.1.1.7, BA.1.1.8<br>BA.1.1.9, BA.1.1.10<br>BA.1.1.11, BA.1.1.12<br>BA.1.1.13, BA.1.1.14<br>BA.1.1.15, BA.1.1.16<br>BA.1.1.17, BA.1.1.18<br>BA.1.2, BA.1.3, BA.1.5,<br>BA.1.6, BA.1.7, BA.1.8,<br>BA.1.9, BA.1.10, BA.1.13,<br>BA.1.13.1, BA.1.14,<br>BA.1.14.1, BA.1.14.2,<br>BA.1.15, BA.1.15.1,<br>BA.1.15.2, BA.1.15.3,<br>BA.1.16, BA.1.16.2,<br>BA.1.17, BA.1.17.1,<br>BA.1.17.2, BA.1.18,<br>BA.1.19, BA.1.20,<br>BA.1.21.1, BA.1.22,<br>BA.1.23, BA.1.24, BC.1,<br>BC.2, BD.1	BA.1, BA.1.1, BA.1.1.1,<br>BA.1.1.2, BA.1.1.3, BA.1.1.4<br>BA.1.1.5, BA.1.1.6, BA.1.1.7<br>BA.1.1.8, BA.1.1.9, BA.1.1.10<br>BA.1.1.11, BA.1.1.12,<br>BA.1.1.13<br>BA.1.1.14, BA.1.1.15,<br>BA.1.1.16<br>BA.1.1.17, BA.1.1.18, BA.1.2<br>BA.1.3, BA.1.5, BA.1.6,<br>BA.1.7<br>BA.1.8, BA.1.9, BA.1.10,<br>BA.1.12, BA.1.13, BA.1.13.1<br>BA.1.14, BA.1.14.1, BA.1.14.2<br>BA.1.15, BA.1.15.1, BA.1.15.2<br>BA.1.15.3, BA.1.16, BA.1.16.1<br>BA.1.16.2, BA.1.17, BA.1.17.1<br>BA.1.17.2, BA.1.18, BA.1.19<br>BA.1.20, BA.1.21.1, BA.1.22<br>BA.1.23, BA.1.24, BC.1, BC.2<br>BD.1
Omicron		BA.2	BA.2, BA.2.1, BA.2.2<br>BA.2.3, BA.2.3.2<br>BA.2.3.4, BA.2.3.5<br>BA.2.3.6, BA.2.3.7<br>BA.2.3.9, BA.2.3.10<br>BA.2.3.11, BA.2.3.15<br>BA.2.3.20, BA.2.4,<br>BA.2.5, BA.2.6, BA.2.7<br>BA.2.8, BA.2.9,<br>BA.2.9.1, BA.2.9.2<br>BA.2.9.3, BA.2.9.4<br>BA.2.9.5, BA.2.9.6<br>BA.2.9.7, BA.2.10<br>BA.2.10.1, BA.2.10.2<br>BA.2.11, BA.2.12<br>BA.2.12.1, BA.2.12.2<br>BA.2.13, BA.2.13.1	BA.2, BA.2.1,<br>BA.2.2, BA.2.3<br>BA.2.3.2, BA.2.3.4<br>BA.2.3.5, BA.2.3.6<br>BA.2.3.7, BA.2.3.8<br>BA.2.3.9, BA.2.3.10,<br>BA.2.3.11, BA.2.3.12<br>BA.2.3.14, BA.2.3.15<br>BA.2.3.16, BA.2.3.17<br>BA.2.3.20, BA.2.3.22<br>BA.2.4, BA.2.5,<br>BA.2.6, BA.2.7<br>BA.2.8, BA.2.9<br>BA.2.9.1, BA.2.9.2<br>BA.2.9.3, BA.2.9.4<br>BA.2.9.5, BA.2.9.6<br>BA.2.9.7, BA.2.10	BA.2, BA.2.1, BA.2.2,<br>BA.2.3, BA.2.3.1,<br>BA.2.3.2, BA.2.3.5,<br>BA.2.3.7, BA.2.3.8,<br>BA.2.3.11, BA.2.3.12,<br>BA.2.3.13, BA.2.3.14,<br>BA.2.3.15, BA.2.3.16,<br>BA.2.3.17, BA.2.3.20,<br>BA.2.3.21, BA.2.4,<br>BA.2.5, BA.2.8,<br>BA.2.9, BA.2.9.3,<br>BA.2.9.5, BA.2.9.6<br>BA.2.9.7, BA.2.10<br>BA.2.10.1, BA.2.11<br>BA.2.12, BA.2.12.1<br>BA.2.12.2, BA.2.13<br>BA.2.13.1, BA.2.16	BA.2, BA.2.1,<br>BA.2.3, BA.2.5<br>BA.2.7, BA.2.8<br>BA.2.9, BA.2.9.7<br>BA.2.10, BA.2.10.1<br>BA.2.12.1, BA.2.15<br>BA.2.16, BA.2.18<br>BA.2.19, BA.2.23<br>BA.2.26, BA.2.36<br>BA.2.37, BA.2.38.2<br>BA.2.38.3,<br>BA.2.40.1, BA.2.45<br>BA.2.50, BA.2.53<br>BA.2.62, BA.2.72<br>BA.2.74, BA.2.76<br>BA.2.78, BA.2.85<br>BG.2, BG.3, CM.4	BA.2, BA.2.1, BA.2.2<br>BA.2.2.1, BA.2.3,<br>BA.2.3.1, BA.2.3.2<br>BA.2.3.4, BA.2.3.5<br>BA.2.3.6, BA.2.3.7<br>BA.2.3.9, BA.2.3.10<br>BA.2.3.11, BA.2.3.12<br>BA.2.3.13, BA.2.3.14<br>BA.2.3.15, BA.2.3.16<br>BA.2.3.17, BA.2.3.19<br>BA.2.3.20, BA.2.4,<br>BA.2.5, BA.2.6, BA.2.7<br>BA.2.8, BA.2.9,<br>BA.2.9.1, BA.2.9.2<br>BA.2.9.3, BA.2.9.4<br>BA.2.9.5, BA.2.9.6<br>BA.2.9.7, BA.2.10	BA.2, BA.2.1, BA.2.2<br>BA.2.2.1, BA.2.3, BA.2.3.1<br>BA.2.3.2, BA.2.3.4,<br>BA.2.3.5, BA.2.3.6,<br>BA.2.3.7, BA.2.3.8,<br>BA.2.3.9, BA.2.3.10,<br>BA.2.3.11, BA.2.3.12,<br>BA.2.3.13, BA.2.3.14,<br>BA.2.3.15, BA.2.3.16,<br>BA.2.3.17, BA.2.3.19,<br>BA.2.3.20, BA.2.3.21,<br>BA.2.4, BA.2.5, BA.2.6,<br>BA.2.7, BA.2.8, BA.2.9,<br>BA.2.9.1, BA.2.9.2,<br>BA.2.9.3, BA.2.9.4,<br>BA.2.9.5, BA.2.9.6,<br>BA.2.9.7, BA.2.10,	BA.2, BA.2.1, BA.2.2,<br>BA.2.2.1, BA.2.3, BA.2.3.1,<br>BA.2.3.2, BA.2.3.4, BA.2.3.5,<br>BA.2.3.6, BA.2.3.7, BA.2.3.8,<br>BA.2.3.9, BA.2.3.10,<br>BA.2.3.11, BA.2.3.12,<br>BA.2.3.13, BA.2.3.14,<br>BA.2.3.15, BA.2.3.16,<br>BA.2.3.17, BA.2.3.18,<br>BA.2.3.19, BA.2.3.20,<br>BA.2.3.21, BA.2.3.22, BA.2.4,<br>BA.2.5, BA.2.6, BA.2.7,<br>BA.2.8, BA.2.9, BA.2.9.1,<br>BA.2.9.2, BA.2.9.3, BA.2.9.4,<br>BA.2.9.5, BA.2.9.6, BA.2.9.7,<br>BA.2.10, BA.2.10.1, BA.2.10.2,<br>BA.2.10.3, BA.2.10.4, BA.2.11,

			BA.2.14, BA.2.15, BA.2.16, BA.2.17 BA.2.18, BA.2.19 BA.2.20, BA.2.21 BA.2.22, BA.2.23 BA.2.23.1, BA.2.24 BA.2.25, BA.2.26, BA.2.27, BA.2.28 BA.2.29, BA.2.31 BA.2.31.1, BA.2.32 BA.2.34, BA.2.35 BA.2.36, BA.2.37 BA.2.38, BA.2.38.1 BA.2.38.3, BA.2.38.4 BA.2.39, BA.2.40 BA.2.40.1, BA.2.41 BA.2.42, BA.2.43 BA.2.44, BA.2.45 BA.2.47, BA.2.48 BA.2.49, BA.2.50 BA.2.51, BA.2.52 BA.2.53, BA.2.54 BA.2.55, BA.2.56 BA.2.58, BA.2.61 BA.2.62, BA.2.63, BA.2.65, BA.2.67 BA.2.70, BA.2.71 BA.2.72, BA.2.73 BA.2.74, BA.2.76 BA.2.79, BA.2.80 BA.2.81, BA.2.82 BA.2.83, BG.2, BG.4 BG.5, BH.1, BJ.1, BS.1 BS.1.1, BS.1.2, CM.1 CM.2, CM.3, CM.4	BA.2.10.1, BA.2.10.2 BA.2.10.3, BA.2.10.4 BA.2.11, BA.2.12 BA.2.12.1, BA.2.12.2 BA.2.13, BA.2.13.1 BA.2.14, BA.2.15 BA.2.16, BA.2.18 BA.2.19, BA.2.20 BA.2.21, BA.2.22 BA.2.23, BA.2.23.1 BA.2.24, BA.2.25 BA.2.25.1, BA.2.26 BA.2.27, BA.2.28 BA.2.29, BA.2.30 BA.2.31, BA.2.31.1 BA.2.32, BA.2.33 BA.2.34, BA.2.35 BA.2.36, BA.2.37 BA.2.38, BA.2.38.1 BA.2.38.2, BA.2.38.3 BA.2.39, BA.2.40 BA.2.40.1, BA.2.41 BA.2.42, BA.2.43 BA.2.44, BA.2.45 BA.2.46, BA.2.47 BA.2.48, BA.2.49 BA.2.50, BA.2.51 BA.2.52, BA.2.53 BA.2.54, BA.2.55 BA.2.56, BA.2.57 BA.2.58, BA.2.60 BA.2.61, BA.2.62 BA.2.63, BA.2.65 BA.2.66, BA.2.67 BA.2.68, BA.2.70 BA.2.71, BA.2.72 BA.2.73, BA.2.74 BA.2.76, BA.2.77 BA.2.78, BA.2.79 BA.2.79.1, BA.2.81 BA.2.82, BA.2.85 BG.2, BG.4, BG.5 BH.1, CM.1, CM.11 CM.2, CM.4, CM.5	BA.2.17, BA.2.18 BA.2.20, BA.2.21 BA.2.23, BA.2.24 BA.2.26, BA.2.27 BA.2.29, BA.2.31.1 BA.2.32, BA.2.36 BA.2.38, BA.2.38.1 BA.2.38.2, BA.2.38.3 BA.2.39, BA.2.40.1 BA.2.41, BA.2.42 BA.2.43, BA.2.46 BA.2.48, BA.2.50 BA.2.52, BA.2.56 BA.2.57, BA.2.58 BA.2.60, BA.2.61 BA.2.62, BA.2.63 BA.2.64, BA.2.65 BA.2.68, BA.2.71 BA.2.72, BA.2.73 BA.2.74, BA.2.76 BA.2.78, BA.2.79 BA.2.81, BA.2.82 BG.2, BG.4, BG.5 BH.1, BJ.1, BP.1 BS.1, BS.1.1, BS.1.2 CM.1, CM.2, CM.3 CM.4, CM.4.1, CM.5 CM.5.1, CM.6, CM.7 CM.8.1		BA.2.10.1, BA.2.10.2 BA.2.10.3, BA.2.10.4 BA.2.11, BA.2.12, BA.2.12.1, BA.2.12.2 BA.2.13, BA.2.13.1 BA.2.14, BA.2.15 BA.2.16, BA.2.17 BA.2.18, BA.2.20 BA.2.21, BA.2.22 BA.2.23, BA.2.23.1 BA.2.24, BA.2.25, BA.2.26, BA.2.27 BA.2.28, BA.2.29 BA.2.30, BA.2.31 BA.2.31.1, BA.2.32 BA.2.33, BA.2.34, BA.2.35, BA.2.36 BA.2.37, BA.2.38 BA.2.38.1, BA.2.38.2 BA.2.38.3, BA.2.39 BA.2.40, BA.2.40.1 BA.2.41, BA.2.42, BA.2.43, BA.2.44 BA.2.45, BA.2.46 BA.2.47, BA.2.48 BA.2.49, BA.2.50 BA.2.51, BA.2.52 BA.2.53, BA.2.54 BA.2.55, BA.2.56 BA.2.57, BA.2.58 BA.2.60, BA.2.61 BA.2.62, BA.2.63, BA.2.64, BA.2.65 BA.2.66, BA.2.67 BA.2.68, BA.2.70 BA.2.71, BA.2.72 BA.2.73, BA.2.74 BA.2.76, BA.2.77 BA.2.78, BA.2.79 BA.2.80, BA.2.81 BA.2.82, BG.1, BG.2 BG.4, BG.5, BG.6, BH.1 BJ.1, BS.1, BS.1.1, CM.2, CM.4, CM.5	BA.2.10.1, ,BA.2.10.2, BA.2.10.3, BA.2.10.4, BA.2.11, BA.2.12, BA.2.12.1, BA.2.12.2, BA.2.13, BA.2.13.1, BA.2.14, BA.2.15, BA.2.16, BA.2.17, BA.2.18, BA.2.19, BA.2.20, BA.2.21, BA.2.22, BA.2.23, BA.2.23.1, BA.2.24, BA.2.25, BA.2.25.1, BA.2.26, BA.2.27, BA.2.28, BA.2.29, BA.2.30, BA.2.31, BA.2.31.1, BA.2.32, BA.2.33, BA.2.34, BA.2.35, BA.2.36, BA.2.37, BA.2.38, BA.2.38.1, BA.2.38.2, BA.2.38.3, BA.2.38.4, BA.2.39, BA.2.40, BA.2.40.1, BA.2.41, BA.2.42, BA.2.43, BA.2.44, BA.2.45, BA.2.47, BA.2.48, BA.2.49, BA.2.50, BA.2.51, BA.2.52, BA.2.53, BA.2.54, BA.2.55, BA.2.56, BA.2.57, BA.2.58, BA.2.59, BA.2.60, BA.2.61, BA.2.62, BA.2.63, BA.2.64, BA.2.65, BA.2.66, BA.2.68, BA.2.69, BA.2.70, BA.2.71, BA.2.72, BA.2.73, BA.2.74, BA.2.76, BA.2.76.1, BA.2.77, BA.2.78, BA.2.79, BA.2.79.1, BA.2.81, BA.2.82, BA.2.85, BG.1, BG.2, BG.3, BG.4, BG.5, BG.6, BG.7, BH.1, BJ.1, BP.1, BS.1, BS.1.1, CM.1, CM.11, CM.12, CM.2, CM.3, CM.4, CM.4.1, CM.5, CM.5.1, CM.5.2, CM.6, CM.6.1, CM.7, CM.8, CM.8.1, CM.9, DD.1	BA.2.12, BA.2.12.1, BA.2.12.2, BA.2.13, BA.2.13.1, BA.2.14, BA.2.15, BA.2.16, BA.2.17, BA.2.18, BA.2.19, BA.2.20, BA.2.21, BA.2.22, BA.2.23, BA.2.23.1, BA.2.24, BA.2.25, BA.2.25.1, BA.2.26, BA.2.27, BA.2.28, BA.2.29, BA.2.30, BA.2.31, BA.2.31.1, BA.2.32, BA.2.33, BA.2.34, BA.2.35, BA.2.36, BA.2.37, BA.2.38, BA.2.38.1, BA.2.38.2, BA.2.38.3, BA.2.38.4, BA.2.39, BA.2.40, BA.2.40.1, BA.2.41, BA.2.42, BA.2.43, BA.2.44, BA.2.45, BA.2.46, BA.2.47, BA.2.48, BA.2.49, BA.2.50, BA.2.51, BA.2.52, BA.2.53, BA.2.54, BA.2.55, BA.2.56, BA.2.56.1, BA.2.57, BA.2.58, BA.2.59, BA.2.60, BA.2.61, BA.2.62, BA.2.63, BA.2.64, BA.2.65, BA.2.66, BA.2.67, BA.2.68, BA.2.69, BA.2.70, BA.2.71, BA.2.72, BA.2.73, BA.2.74, BA.2.76, BA.2.76.1, BA.2.76.2, BA.2.77, BA.2.78, BA.2.79, BA.2.79.1, BA.2.80, BA.2.81, BA.2.82, BA.2.83, BA.2.85, BG.1, BG.2, BG.3, BG.4, BG.5, BG.6, BG.7, BH.1, BJ.1, BP.1, BS.1, BS.1.1, BS.1.2, CM.1, CM.2, CM.2.1, CM.3, CM.4, CM.4.1, CM.5, CM.5.1, CM.5.2, CM.6, CM.6.1, CM.7, CM.8, CM.8.1, CM.9, CM.10, CM.11, CM.12, DD.1
Omicron		BA.3	BA.3, BA.3.1	BA.3, BA.3.1		BA.3	BA.3, BA.3.1	BA.3, BA.3.1	BA.3, BA.3.1
Omicron		BA.4	BA.4, BA.4.1, BA.4.1.1, BA.4.1.2, BA.4.1.3, BA.4.1.4, BA.4.1.5, BA.4.1.6, BA.4.1.7, BA.4.1.8,	BA.4, BA.4.1, BA.4.1.1, BA.4.1.2, BA.4.1.3, BA.4.1.4, BA.4.1.5, BA.4.1.6, BA.4.1.7, BA.4.1.8,	BA.4, BA.4.1, BA.4.1.1, BA.4.1.4, BA.4.1.6, BA.4.1.7, BA.4.1.8, BA.4.1.9, BA.4.1.10, BA.4.2,	BA.4, BA.4.1, BA.4.1.1, BA.4.1.2, BA.4.1.7, BA.4.1.8, BA.4.1.9, BA.4.1.10, BA.4.2,	BA.4, BA.4.1, BA.4.1.1 BA.4.1.3, BA.4.1.4, BA.4.1.5, BA.4.1.6, BA.4.1.7, BA.4.1.8, BA.4.1.9, BA.4.1.10,	BA.4, BA.4.1, BA.4.1.1, BA.4.1.2, BA.4.1.3, BA.4.1.4, BA.4.1.5, BA.4.1.6, BA.4.1.7, BA.4.1.8, BA.4.1.9,	BA.4, BA.4.1, BA.4.1.1, BA.4.1.2, BA.4.1.3, BA.4.1.4, BA.4.1.5, BA.4.1.6, BA.4.1.7, BA.4.1.8, BA.4.1.9, BA.4.1.10, BA.4.2, BA.4.3, BA.4.4,

			BA.4.1.9, BA.4.1.10,<br>BA.4.2, BA.4.3,<br>BA.4.4, BA.4.5,<br>BA.4.6, BA.4.6.1,<br>BA.4.6.2, BA.4.6.3,<br>BA.4.6.4, BA.4.6.5,<br>BA.4.7, CS.1, DC.1	BA.4.1.9, BA.4.1.10,<br>BA.4.2, BA.4.3,<br>BA.4.4, BA.4.5,<br>BA.4.6, BA.4.6.1,<br>BA.4.6.2, BA.4.6.3,<br>BA.4.6.4, BA.4.6.5,<br>BA.4.7, DC.1	BA.4.3, BA.4.4,<br>BA.4.5, BA.4.6,<br>BA.4.6.2, BA.4.6.3,<br>BA.4.6.4, BA.4.6.5,<br>BA.4.7	BA.4.3, BA.4.4,<br>BA.4.5, BA.4.6,<br>BA.4.6.3, BA.4.6.5,<br>BA.4.7	BA.4.2, BA.4.3, BA.4.4,<br>BA.4.5, BA.4.6,<br>BA.4.6.1, BA.4.6.2,<br>BA.4.6.3, BA.4.6.4,<br>BA.4.6.5, BA.4.7, CS.1,<br>DC.1	BA.4.1.10, BA.4.2, BA.4.3,<br>BA.4.4, BA.4.5, BA.4.6,<br>BA.4.6.1, BA.4.6.2,<br>BA.4.6.3, BA.4.6.4,<br>BA.4.6.5, BA.4.7, CS.1,<br>DC.1	BA.4.5, BA.4.6, BA.4.6.1,<br>BA.4.6.2, BA.4.6.3, BA.4.6.4,<br>BA.4.6.5, BA.4.7, CS.1, DC.1
Omicron		BA.5	BA.5, BA.5.1, BA.5.1.1<br>BA.5.1.2, BA.5.1.3<br>BA.5.1.4, BA.5.1.5<br>BA.5.1.6, BA.5.1.7<br>BA.5.1.8, BA.5.1.9<br>BA.5.1.10, BA.5.1.11<br>BA.5.1.12, BA.5.1.15<br>BA.5.1.16, BA.5.1.17<br>BA.5.1.18, BA.5.1.19<br>BA.5.1.20, BA.5.1.21<br>BA.5.1.22, BA.5.1.23<br>BA.5.1.24, BA.5.1.25<br>BA.5.1.26, BA.5.1.27<br>BA.5.1.28, BA.5.1.30<br>BA.5.1.31, BA.5.1.32<br>BA.5.2, BA.5.2.1<br>BA.5.2.2, BA.5.2.3<br>BA.5.2.4, BA.5.2.5<br>BA.5.2.6, BA.5.2.7<br>BA.5.2.8, BA.5.2.9<br>BA.5.2.10, BA.5.2.11<br>BA.5.2.12, BA.5.2.13<br>BA.5.2.14, BA.5.2.16<br>BA.5.2.18, BA.5.2.19<br>BA.5.2.20, BA.5.2.21<br>BA.5.2.22, BA.5.2.23<br>BA.5.2.24, BA.5.2.25<br>BA.5.2.26, BA.5.2.27<br>BA.5.2.28, BA.5.2.29<br>BA.5.2.30, BA.5.2.31<br>BA.5.2.32, BA.5.2.33<br>BA.5.2.34, BA.5.2.35<br>BA.5.2.36, BA.5.2.37<br>BA.5.2.38, BA.5.2.41<br>BA.5.2.44, BA.5.2.46<br>BA.5.2.47, BA.5.2.48<br>BA.5.3, BA.5.3.1<br>BA.5.3.2, BA.5.3.3<br>BA.5.3.4, BA.5.5<br>BA.5.5.1, BA.5.5.2<br>BA.5.5.3, BA.5.6<br>BA.5.6.1, BA.5.6.2<br>BA.5.6.3, BA.5.6.4<br>BA.5.7, BA.5.8, BA.5.9	BA.5, BA.5.1,<br>BA.5.1.1, BA.5.1.2<br>BA.5.1.3, BA.5.1.4<br>BA.5.1.5, BA.5.1.6<br>BA.5.1.7, BA.5.1.8<br>BA.5.1.9, BA.5.1.10<br>BA.5.1.11, BA.5.1.12<br>BA.5.1.15, BA.5.1.16<br>BA.5.1.17, BA.5.1.18<br>BA.5.1.19, BA.5.1.20<br>BA.5.1.21, BA.5.1.22<br>BA.5.1.23, BA.5.1.24<br>BA.5.1.25, BA.5.1.26<br>BA.5.1.27, BA.5.1.28<br>BA.5.1.29, BA.5.1.30<br>BA.5.1.31, BA.5.1.32<br>BA.5.2, BA.5.2.1<br>BA.5.2.2, BA.5.2.3<br>BA.5.2.4, BA.5.2.5<br>BA.5.2.6, BA.5.2.7<br>BA.5.2.8, BA.5.2.9<br>BA.5.2.10, BA.5.2.12<br>BA.5.2.13, BA.5.2.14<br>BA.5.2.16, BA.5.2.18<br>BA.5.2.19, BA.5.2.20<br>BA.5.2.21, BA.5.2.22<br>BA.5.2.23, BA.5.2.24<br>BA.5.2.25, BA.5.2.26<br>BA.5.2.27, BA.5.2.28<br>BA.5.2.30, BA.5.2.31<br>BA.5.2.32, BA.5.2.33<br>BA.5.2.34, BA.5.2.35<br>BA.5.2.36, BA.5.2.37<br>BA.5.2.38, BA.5.2.39<br>BA.5.2.40, BA.5.2.41<br>BA.5.2.44, BA.5.2.47<br>BA.5.2.48, BA.5.3<br>BA.5.3.1, BA.5.3.2<br>BA.5.3.3, BA.5.3.4<br>BA.5.5, BA.5.5.1<br>BA.5.5.2, BA.5.5.3<br>BA.5.6, BA.5.6.1<br>BA.5.6.2, BA.5.6.3<br>BA.5.6.4, BA.5.7	BA.5, BA.5.1,<br>BA.5.1.1, BA.5.1.2<br>BA.5.1.3, BA.5.1.4<br>BA.5.1.5, BA.5.1.6<br>BA.5.1.7, BA.5.1.8<br>BA.5.1.9, BA.5.1.10<br>BA.5.1.11, BA.5.1.12<br>BA.5.1.15, BA.5.1.16<br>BA.5.1.17, BA.5.1.19<br>BA.5.1.21, BA.5.1.22<br>BA.5.1.23, BA.5.1.24<br>BA.5.1.25, BA.5.1.26<br>BA.5.1.28, BA.5.1.29<br>BA.5.1.30, BA.5.1.31<br>BA.5.1.32, BA.5.2<br>BA.5.2.1, BA.5.2.2<br>BA.5.2.3, BA.5.2.4<br>BA.5.2.5, BA.5.2.6<br>BA.5.2.7, BA.5.2.9<br>BA.5.2.11, BA.5.2.12<br>BA.5.2.13, BA.5.2.14<br>BA.5.2.16, BA.5.2.18<br>BA.5.2.19, BA.5.2.20<br>BA.5.2.21, BA.5.2.22<br>BA.5.2.23, BA.5.2.24<br>BA.5.2.25, BA.5.2.26<br>BA.5.2.27, BA.5.2.28<br>BA.5.2.30, BA.5.2.31<br>BA.5.2.32, BA.5.2.33<br>BA.5.2.34, BA.5.2.35<br>BA.5.2.36, BA.5.2.37<br>BA.5.2.38, BA.5.2.43<br>BA.5.2.44, BA.5.2.45<br>BA.5.2.47, BA.5.2.48<br>BA.5.2.49, BA.5.3<br>BA.5.3.1, BA.5.3.2<br>BA.5.3.3, BA.5.3.4<br>BA.5.5, BA.5.5.1<br>BA.5.5.2, BA.5.6<br>BA.5.6.2, BA.5.6.4<br>BA.5.7, BA.5.8,<br>BA.5.9, BA.5.10<br>BA.5.10.1, BE.1,<br>BE.1.1, BE.1.1.1	BA.5, BA.5.1<br>BA.5.1.2, BA.5.1.3<br>BA.5.1.8, BA.5.1.9<br>BA.5.1.10,<br>BA.5.1.17,<br>BA.5.1.18,<br>BA.5.1.20,<br>BA.5.1.21,<br>BA.5.1.22,<br>BA.5.1.23,<br>BA.5.1.24, BA.5.2<br>BA.5.2.1, BA.5.2.3<br>BA.5.2.6, BA.5.2.7<br>BA.5.2.13,<br>BA.5.2.16,<br>BA.5.2.18,<br>BA.5.2.20,<br>BA.5.2.22,<br>BA.5.2.24,<br>BA.5.2.27,<br>BA.5.2.28,<br>BA.5.2.37, BA.5.3<br>BA.5.3.1, BA.5.3.3<br>BA.5.3.5, BA.5.5<br>BA.5.6, BA.5.8<br>BA.5.9, BA.5.10<br>BA.5.10.1, BA.5.11<br>BE.1, BE.1.1,<br>BE.1.1.1, BE.1.1.2<br>BE.2, BE.3, BE.6<br>BE.7, BE.8, BE.9<br>BE.10, BF.2, BF.3<br>BF.4, BF.5, BF.7<br>BF.7.8, BF.9, BF.10<br>BF.14, BF.15, BF.17<br>BF.23, BF.24, BF.25<br>BF.26, BF.27, BF.28<br>BF.30, BF.31, BQ.1<br>BQ.1.1, BQ.1.1.38<br>BQ.1.3, BQ.1.5,<br>BQ.1.6, BQ.1.13<br>BQ.1.22, BQ.1.23<br>BQ.1.24, BU.1<br>CK.2.1.1, CP.5	BA.5, BA.5.1, BA.5.1.1<br>BA.5.1.2, BA.5.1.3<br>BA.5.1.4, BA.5.1.5<br>BA.5.1.6, BA.5.1.7<br>BA.5.1.8, BA.5.1.9<br>BA.5.1.10, BA.5.1.11<br>BA.5.1.12, BA.5.1.15<br>BA.5.1.16, BA.5.1.17<br>BA.5.1.18, BA.5.1.19<br>BA.5.1.20, BA.5.1.21<br>BA.5.1.22, BA.5.1.23<br>BA.5.1.24, BA.5.1.25<br>BA.5.1.26, BA.5.1.27<br>BA.5.1.28, BA.5.1.30<br>BA.5.1.31, BA.5.1.32<br>BA.5.2, BA.5.2.1<br>BA.5.2.2, BA.5.2.3<br>BA.5.2.4, BA.5.2.6<br>BA.5.2.7, BA.5.2.8<br>BA.5.2.9, BA.5.2.10<br>BA.5.2.12, BA.5.2.13<br>BA.5.2.14, BA.5.2.16<br>BA.5.2.18, BA.5.2.19<br>BA.5.2.20, BA.5.2.21<br>BA.5.2.22, BA.5.2.23<br>BA.5.2.24, BA.5.2.25<br>BA.5.2.26, BA.5.2.27<br>BA.5.2.28, BA.5.2.29<br>BA.5.2.30, BA.5.2.31<br>BA.5.2.32, BA.5.2.33<br>BA.5.2.34, BA.5.2.35<br>BA.5.2.36, BA.5.2.37<br>BA.5.2.38, BA.5.2.39<br>BA.5.2.40, BA.5.2.41<br>BA.5.2.43, BA.5.2.44<br>BA.5.2.47, BA.5.3<br>BA.5.3.1, BA.5.3.2<br>BA.5.3.3, BA.5.3.4<br>BA.5.3.5, BA.5.5<br>BA.5.5.1, BA.5.5.2<br>BA.5.5.3, BA.5.6<br>BA.5.6.1, BA.5.6.2<br>BA.5.6.3, BA.5.6.4<br>BA.5.7, BA.5.8, BA.5.9	BA.5, BA.5.1, BA.5.1.1<br>BA.5.1.2, BA.5.1.3<br>BA.5.1.4, BA.5.1.5<br>BA.5.1.6, BA.5.1.7<br>BA.5.1.8, BA.5.1.9<br>BA.5.1.10, BA.5.1.11<br>BA.5.1.12, BA.5.1.15<br>BA.5.1.16, BA.5.1.17<br>BA.5.1.18, BA.5.1.19<br>BA.5.1.20, BA.5.1.21<br>BA.5.1.22, BA.5.1.23<br>BA.5.1.24, BA.5.1.25<br>BA.5.1.26, BA.5.1.27<br>BA.5.1.28, BA.5.1.29<br>BA.5.1.30, BA.5.1.31<br>BA.5.1.32, BA.5.2<br>BA.5.2.1, BA.5.2.2,<br>BA.5.2.3, BA.5.2.4,<br>BA.5.2.5, BA.5.2.6<br>BA.5.2.7, BA.5.2.8<br>BA.5.2.9, BA.5.2.10<br>BA.5.2.11, BA.5.2.12<br>BA.5.2.13, BA.5.2.14<br>BA.5.2.16, BA.5.2.18<br>BA.5.2.19, BA.5.2.20<br>BA.5.2.21, BA.5.2.22<br>BA.5.2.23, BA.5.2.24<br>BA.5.2.25, BA.5.2.26<br>BA.5.2.27, BA.5.2.28<br>BA.5.2.29, BA.5.2.30<br>BA.5.2.31, BA.5.2.32<br>BA.5.2.33, BA.5.2.34<br>BA.5.2.35, BA.5.2.36<br>BA.5.2.37, BA.5.2.38<br>BA.5.2.39, BA.5.2.41<br>BA.5.2.43, BA.5.2.44<br>BA.5.2.45, BA.5.2.46<br>BA.5.2.47, BA.5.2.49<br>BA.5.3, BA.5.3.1, BA.5.3.2<br>BA.5.3.3, BA.5.3.4,<br>BA.5.3.5, BA.5.5, BA.5.5.1<br>BA.5.5.2, BA.5.5.3, BA.5.6<br>BA.5.6.1, BA.5.6.2,<br>BA.5.6.3, BA.5.6.4, BA.5.7	BA.5, BA.5.1, BA.5.1.1<br>BA.5.1.2, BA.5.1.3, BA.5.1.4<br>BA.5.1.5, BA.5.1.6, BA.5.1.7<br>BA.5.1.8, BA.5.1.9, BA.5.1.10<br>BA.5.1.11, BA.5.1.12,<br>BA.5.1.14, BA.5.1.15<br>BA.5.1.16, BA.5.1.17<br>BA.5.1.18, BA.5.1.19<br>BA.5.1.20, BA.5.1.21<br>BA.5.1.22, BA.5.1.23<br>BA.5.1.24, BA.5.1.25<br>BA.5.1.26, BA.5.1.27<br>BA.5.1.28, BA.5.1.29<br>BA.5.1.30, BA.5.1.31<br>BA.5.1.32, BA.5.2, BA.5.2.1<br>BA.5.2.2, BA.5.2.3, BA.5.2.4<br>BA.5.2.5, BA.5.2.6, BA.5.2.7<br>BA.5.2.8, BA.5.2.9, BA.5.2.10<br>BA.5.2.11, BA.5.2.12,<br>BA.5.2.13, BA.5.2.14<br>BA.5.2.16, BA.5.2.18<br>BA.5.2.19, BA.5.2.20<br>BA.5.2.21, BA.5.2.22<br>BA.5.2.23, BA.5.2.24<br>BA.5.2.25, BA.5.2.26<br>BA.5.2.27, BA.5.2.28<br>BA.5.2.29, BA.5.2.30<br>BA.5.2.31, BA.5.2.32<br>BA.5.2.33, BA.5.2.34<br>BA.5.2.35, BA.5.2.36<br>BA.5.2.37, BA.5.2.38<br>BA.5.2.39, BA.5.2.40<br>BA.5.2.41, BA.5.2.42<br>BA.5.2.43, BA.5.2.44<br>BA.5.2.45, BA.5.2.46<br>BA.5.2.47, BA.5.2.48<br>BA.5.2.49, BA.5.2.50<br>BA.5.3, BA.5.3.1, BA.5.3.2<br>BA.5.3.3, BA.5.3.4, BA.5.3.5<br>BA.5.5, BA.5.5.1, BA.5.5.2<br>BA.5.5.3, BA.5.6, BA.5.6.1<br>BA.5.6.2, BA.5.6.3, BA.5.6.4<br>BA.5.7, BA.5.8, BA.5.9,<br>BA.5.10, BA.5.10.1, BA.5.11

			BA.5.10, BA.5.10.1 BA.5.11, BE.1, BE.1.1 BE.1.1.1, BE.1.1.2 BE.1.2, BE.1.2.1, BE.1.3, BE.1.4, BE.1.4.1, BE.2, BE.3 BE.4, BE.4.1, BE.6, BE.7, BE.8, BE.9, BF.1 BF.1.1, BF.2, BF.3 BF.3.1, BF.4, BF.5, BF.5.1, BF.6, BF.7, BF.7.1, BF.7.2, BF.7.3 BF.7.4, BF.7.4.1, BF.7.4.2, BF.7.5, BF.7.5.1, BF.7.6, BF.7.7, BF.7.8, BF.7.9 BF.7.10, BF.7.11, BF.7.12, BF.7.13, BF.7.13.1, BF.7.13.2, BF.8, BF.9, BF.10, BF.11, BF.11.1, BF.11.2, BF.11.3, BF.11.4, BF.11.5, BF.12, BF.13, BF.14, BF.15, BF.16, BF.17, BF.18, BF.19, BF.20, BF.21, BF.23, BF.24 BF.25, BF.26, BF.27 BF.28, BF.29, BF.30 BF.31, BF.34, BK.1 BQ.1, BQ.1.1, BQ.1.1.1 BQ.1.1.2, BQ.1.1.3 BQ.1.1.4, BQ.1.1.5 BQ.1.1.6, BQ.1.1.7 BQ.1.1.8, BQ.1.1.10 BQ.1.1.11, BQ.1.1.13 BQ.1.1.15, BQ.1.1.17 BQ.1.1.18, BQ.1.1.19 BQ.1.1.20, BQ.1.1.22 BQ.1.1.23, BQ.1.1.27 BQ.1.1.28, BQ.1.1.47 BQ.1.2, BQ.1.3, BQ.1.4 BQ.1.5, BQ.1.8, BQ.1.8.2, BQ.1.10, BQ.1.10.1, BQ.1.11 BQ.1.12, BQ.1.13, BQ.1.14, BQ.1.15 BQ.1.17, BQ.1.18 BQ.1.19, BQ.1.23 BQ.1.25, BQ.1.26 BQ.1.26.1, BQ.1.28	BA.5.8, BA.5.9 BA.5.10, BA.5.10.1 BE.1, BE.1.1, BE.1.1.1, BE.1.1.2 BE.1.2, BE.1.2.1 BE.1.3, BE.1.4 BE.1.4.1, BE.1.4.4 BE.2, BE.3, BE.4 BE.4.1, BE.4.1.1 BE.6, BE.7, BE.9 BE.10, BF.1, BF.1.1 BF.2, BF.3, BF.3.1 BF.4, BF.5, BF.5.1 BF.6, BF.7, BF.7.1 BF.7.3, BF.7.4, BF.7.4.1, BF.7.4.2 BF.7.5, BF.7.5.1 BF.7.6, BF.7.7, BF.7.8, BF.7.9 BF.7.10, BF.7.11 BF.7.12, BF.7.13 BF.7.13.1, BF.7.13.2 BF.7.15, BF.8, BF.9 BF.10, BF.11, BF.11.1, BF.11.2 BF.11.3, BF.11.4 BF.11.5, BF.12 BF.13, BF.14, BF.15 BF.16, BF.17, BF.18 BF.19, BF.20, BF.21 BF.22, BF.23, BF.24 BF.25, BF.26, BF.27 BF.28, BF.29, BF.30 BF.31, BF.31.1, BF.32, BF.34, BK.1 BQ.1, BQ.1.1, BQ.1.1.1, BQ.1.1.3 BQ.1.1.4, BQ.1.1.5 BQ.1.1.6, BQ.1.1.7 BQ.1.1.8, BQ.1.1.10 BQ.1.1.11, BQ.1.1.13 BQ.1.1.15, BQ.1.1.16 BQ.1.1.17, BQ.1.1.18 BQ.1.1.19, BQ.1.1.20 BQ.1.1.22, BQ.1.1.23 BQ.1.2, BQ.1.2.1, BQ.1.3, BQ.1.4, BQ.1.5, BQ.1.8, BQ.1.8.2, BQ.1.10, BQ.1.10.1, BQ.1.11 BQ.1.12, BQ.1.13	BE.1.1.2, BE.1.2 BE.1.2.1, BE.1.3 BE.1.4, BE.1.4.1 BE.1.4.4, BE.2, BE.3 BE.4, BE.4.1, BE.4.1.1 BE.4.2, BE.6, BE.7, BE.9, BE.10, BF.1, BF.2, BF.3, BF.3.1 BF.4, BF.5, BF.5.1 BF.6, BF.7, BF.7.10 BF.7.4, BF.7.4.1, BF.7.4.2, BF.7.5, BF.7.5.1, BF.7.6, BF.7.8, BF.8, BF.9 BF.10, BF.11, BF.11.1 BF.11.5, BF.12, BF.13 BF.14, BF.15, BF.16 BF.17, BF.19, BF.20 BF.21, BF.22, BF.23 BF.24, BF.25, BF.26 BF.27, BF.28, BF.30 BF.31, BF.34, BK.1 BQ.1, BQ.1.1, BQ.1.1.1, BQ.1.1.3 BQ.1.1.4, BQ.1.1.5 BQ.1.1.7, BQ.1.1.11 BQ.1.1.17, BQ.1.1.18 BQ.1.1.23, BQ.1.1.26 BQ.1.1.41, BQ.1.1.44 BQ.1.2, BQ.1.2.1 BQ.1.3, BQ.1.5, BQ.1.6, BQ.1.8, BQ.1.9, BQ.1.10 BQ.1.10.1, BQ.1.11 BQ.1.13, BQ.1.14 BQ.1.15, BQ.1.16 BQ.1.17, BQ.1.22 BQ.1.23, BQ.1.24 BQ.1.25, BQ.1.28 BT.1, BT.2, BV.2, BZ.1, CC.1, CD.2 CG.1, CK.1, CK.1.2 CK.2.1, CK.2.1.1, CK.3, CL.1, CN.1 CP.1, CU.1, CY.1, CZ.1, DA.1, DB.1 DB.2, DE.1, DE.2 DF.1, DG.1, DY.1		BA.5.10, BA.5.10.1 BA.5.11, BE.1, BE.1.1 BE.1.1.1, BE.1.1.2 BE.1.2, BE.1.2.1, BE.1.3, BE.1.4, BE.1.4.1, BE.1.4.4, BE.2, BE.3, BE.4 BE.4.1, BE.4.1.1, BE.4.2, BE.6, BE.7, BE.8, BE.9, BF.1, BF.1.1, BF.2, BF.3, BF.3.1, BF.4, BF.5, BF.5.1, BF.6, BF.7, BF.7.1, BF.7.3, BF.7.4 BF.7.4.1, BF.7.4.2, BF.7.5, BF.7.6, BF.7.7 BF.7.8, BF.7.9, BF.7.10 BF.7.11, BF.7.12, BF.7.13, BF.7.13.1 BF.7.13.2, BF.8, BF.9 BF.10, BF.11, BF.11.1 BF.11.2, BF.11.3, BF.11.4, BF.11.5, BF.12 BF.13, BF.14, BF.15 BF.16, BF.17, BF.18 BF.19, BF.20, BF.21 BF.23, BF.24, BF.25 BF.26, BF.27, BF.28 BF.29, BF.30, BF.31 BF.34, BK.1, BQ.1 BQ.1.1, BQ.1.1.1, BQ.1.1.2, BQ.1.1.3 BQ.1.1.4, BQ.1.1.5 BQ.1.1.6, BQ.1.1.7 BQ.1.1.8, BQ.1.1.9 BQ.1.1.10, BQ.1.1.11 BQ.1.1.13, BQ.1.1.14 BQ.1.1.15, BQ.1.1.16 BQ.1.1.17, BQ.1.1.18 BQ.1.1.19, BQ.1.1.22 BQ.1.1.23, BQ.1.1.24 BQ.1.1.27, BQ.1.1.28 BQ.1.1.41, BQ.1.2 BQ.1.3, BQ.1.4, BQ.1.5 BQ.1.6, BQ.1.7, BQ.1.8 BQ.1.8.1, BQ.1.8.2 BQ.1.10, BQ.1.10.1 BQ.1.11, BQ.1.12 BQ.1.13, BQ.1.13.1 BQ.1.14, BQ.1.15 BQ.1.16, BQ.1.17	BA.5.8, BA.5.9, BA.5.10 BA.5.10.1, BA.5.11, BE.1 BE.1.1, BE.1.1.1, BE.1.1.2 BE.1.2, BE.1.2.1, BE.1.3 BE.1.4, BE.1.4.1, BE.1.4.2 BE.1.4.3, BE.1.4.4, BE.2 BE.3, BE.4, BE.4.1, BE.4.1.1, BE.4.2, BE.6, BE.7, BE.8, BE.9, BE.10 BF.1, BF.1.1, BF.2, BF.3 BF.3.1, BF.4, BF.5, BF.5.1 BF.6, BF.7, BF.7.1, BF.7.3 BF.7.4, BF.7.4.1, BF.7.4.2 BF.7.5, BF.7.5.1, BF.7.6 BF.7.7, BF.7.8, BF.7.9 BF.7.10, BF.7.11, BF.7.12 BF.7.13.2, BF.8, BF.9, BF.10, BF.10.1, BF.11 BF.11.1, BF.11.2, BF.11.3 BF.11.4, BF.11.5, BF.12 BF.13, BF.14, BF.15, BF.16 BF.17, BF.18, BF.19, BF.20 BF.21, BF.22, BF.23, BF.24 BF.25, BF.26, BF.27, BF.28 BF.29, BF.30, BF.31, BF.31.1, BF.32, BF.34, BK.1, BQ.1, BQ.1.1, BQ.1.1.1, BQ.1.1.2 BQ.1.1.3, BQ.1.1.4 BQ.1.1.5, BQ.1.1.6 BQ.1.1.7, BQ.1.1.8 BQ.1.1.10, BQ.1.1.11 BQ.1.1.12, BQ.1.1.13 BQ.1.1.14, BQ.1.1.15 BQ.1.1.17, BQ.1.1.18 BQ.1.1.19, BQ.1.1.22 BQ.1.1.23, BQ.1.1.24 BQ.1.1.25, BQ.1.1.27 BQ.1.1.28, BQ.1.1.29 BQ.1.1.31, BQ.1.1.32 BQ.1.1.34, BQ.1.1.35 BQ.1.1.40, BQ.1.1.41 BQ.1.1.47, BQ.1.2, BQ.1.2.1, BQ.1.3, BQ.1.4 BQ.1.5, BQ.1.6, BQ.1.7 BQ.1.8, BQ.1.8.2, BQ.1.9 BQ.1.10, BQ.1.10.1, BQ.1.11, BQ.1.12, BQ.1.13 BQ.1.13.1, BQ.1.14, BQ.1.15, BQ.1.16, BQ.1.18 BQ.1.19, BQ.1.20, BQ.1.21	BE.1, BE.1.1, BE.1.1.1, BE.1.1.2, BE.1.2, BE.1.2.1 BE.1.3, BE.1.4, BE.1.4.1 BE.1.4.2, BE.1.4.3, BE.1.4.4 BE.2, BE.3, BE.4, BE.4.1 BE.4.1.1, BE.4.2, BE.6, BE.7 BE.8, BE.9, BE.10, BF.1, BF.1.1, BF.2, BF.3, BF.3.1 BF.4, BF.5, BF.5.1, BF.5.2 BF.6, BF.7, BF.7.1, BF.7.2 BF.7.3, BF.7.4, BF.7.4.1, BF.7.4.2, BF.7.5, BF.7.5.1 BF.7.6, BF.7.7, BF.7.8, BF.7.9 BF.7.10, BF.7.11, BF.7.12 BF.7.13, BF.7.13.1, BF.7.13.2 BF.7.14, BF.7.14.3, BF.7.15 BF.8, BF.9, BF.10, BF.10.1 BF.11, BF.11.1, BF.11.2, BF.11.3, BF.11.4, BF.11.5, BF.12, BF.13, BF.14, BF.15 BF.16, BF.17, BF.18, BF.19 BF.20, BF.21, BF.22, BF.23 BF.24, BF.25, BF.26, BF.27 BF.28, BF.29, BF.30, BF.31 BF.31.1, BF.32, BF.33, BF.34 BK.1, BQ.1, BQ.1.1, BQ.1.1.1 BQ.1.1.2, BQ.1.1.3, BQ.1.1.4 BQ.1.1.5, BQ.1.1.6, BQ.1.1.7 BQ.1.1.8, BQ.1.1.9, BQ.1.1.10 BQ.1.1.11, BQ.1.1.12, BQ.1.1.13, BQ.1.1.14 BQ.1.1.15, BQ.1.1.16 BQ.1.1.17, BQ.1.1.18 BQ.1.1.19, BQ.1.1.20 BQ.1.1.21, BQ.1.1.22 BQ.1.1.23, BQ.1.1.24 BQ.1.1.25, BQ.1.1.26 BQ.1.1.27, BQ.1.1.28 BQ.1.1.29, BQ.1.1.30 BQ.1.1.31, BQ.1.1.32 BQ.1.1.34, BQ.1.1.35 BQ.1.1.36, BQ.1.1.38 BQ.1.1.39, BQ.1.1.40 BQ.1.1.41, BQ.1.1.42 BQ.1.1.43, BQ.1.1.44 BQ.1.1.45, BQ.1.1.46 BQ.1.1.47, BQ.1.2, BQ.1.2.1 BQ.1.3, BQ.1.4, BQ.1.5, BQ.1.6, BQ.1.7, BQ.1.8, BQ.1.8.1, BQ.1.8.2, BQ.1.9 BQ.1.10, BQ.1.10.1, BQ.1.11

			BT.1, BT.2, BU.1, BU.2, BV.1, BV.2, BW.1, BZ.1, CD.1, CE.1, CG.1, CK.1, CK.1.2, CK.2, CK.2.1.1 CL.1, CN.1, CP.1, CP.1.1, CP.1.2, CP.1.3 CP.2, CP.4, CP.5, CQ.1 CR.1, CR.1.1, CR.1.3 CU.1, DB.1, DE.1, DE.2, DF.1, DG.1, DH.1, DK.1, DL.1, DN.1.1	BQ.1.13.1, BQ.1.14 BQ.1.15, BQ.1.16 BQ.1.17, BQ.1.18 BQ.1.20, BQ.1.22 BQ.1.23, BQ.1.25 BQ.1.26, BQ.1.26.1 BT.1, BT.2, BU.1, BU.3, BV.1, BV.2 BW.1, BZ.1, CC.1 CE.1, CG.1, CK.1 CK.2, CK.2.1.1, CK.3 CL.1, CN.1, CP.1, CP.2, CP.3, CP.4, CP.5, CQ.1, CR.1 CR.1.1, CR.2, CU.1 CY.1, DB.2, DE.1 DE.2, DG.1, DQ.1			BQ.1.18, BQ.1.19 BQ.1.20, BQ.1.21 BQ.1.22, BQ.1.23 BQ.1.24, BQ.1.25 BQ.1.26, BQ.2, BT.1 BT.2, BU.1, BU.3, BV.1 BV.2, BW.1, BW.1.1 CD.1, CE.1, CG.1, CK.1 CK.2, CK.2.1, CK.2.1.1 CK.3, CL.1, CN.1, CP.1 CP.1.1, CP.1.2, CP.1.3 CP.3, CP.5, CQ.1, CQ.1.1, CR.1, CR.1.1 CR.2, CT.1, CW.1, CZ.1 DA.1, DE.1, DE.2, DF.1 DF.1.1, DG.1, DL.1, DN.1, DN.1.1, DP.1, DQ.1, DW.1	BQ.1.22, BQ.1.23, BQ.1.24 BQ.1.25, BQ.1.25.1, BQ.1.26.1, BQ.1.27, BQ.1.28, BQ.2, BT.1, BT.2 BU.1, BU.2, BU.3, BV.1 BV.2, BW.1, BW.1.1, BZ.1 CC.1, CD.1, CD.2, CE.1 CF.1, CG.1, CK.1. CK.1.2 CK.2. CK.2.1. CK.2.1.1 CK.3. CL.1. CN.1. CN.2 CP.1. CP.1.1. CP.1.2. CP.2 CP.3. CP.4. CP.5. CP.6. CQ.1. CQ.1.1. CQ.2. CR.1 CR.1.1. CR.1.3. CR.2. CT.1 CY.1. DA.1. DB.1. DB.2 DE.1. DE.2. DF.1. DF.1.1 DG.1. DJ.1. DJ.1.1. DJ.1.1.1. DK.1. DL.1. DN.1 DQ.1. DR.1. DW.1	BQ.1.11.1, BQ.1.12, BQ.1.13 BQ.1.13.1, BQ.1.14, BQ.1.15 BQ.1.16, BQ.1.17, BQ.1.18 BQ.1.19, BQ.1.20, BQ.1.21 BQ.1.22, BQ.1.23, BQ.1.24 BQ.1.25, BQ.1.25.1, BQ.1.26 BQ.1.26.1, BQ.1.27, BQ.1.28 BQ.2, BT.1, BT.2, BU.1, BU.2 BU.3, BV.1, BV.2, BW.1, BW.1.1, BZ.1, CC.1, CD.1, CD.2, CE.1, CF.1, CG.1, CK.1 CK.1.1, CK.1.2, CK.2, CK.2.1 CK.2.1.1, CK.3, CL.1, CN.1 CN.2, CP.1, CP.1.1, CP.1.2 CP.1.3, CP.2, CP.3, CP.4, CP.5 CP.6, CQ.1, CQ.1.1, CQ.2, CR.1, CR.1.1, CR.1.2, CR.1.3 CR.2, CT.1, CU.1, CW.1, CY.1 CZ.1, DA.1, DB.1, DB.2, DE.1 DE.2, DF.1, DF.1.1, DG.1, DH.1, DJ.1, DJ.1.1, DJ.1.1.1 DJ.1.2, DJ.1.3, DK.1, DL.1 DM.1, DN.1, DN.1.1, DP.1 DQ.1, DR.1, DW.1, DY.1,DZ.2
Omicron	Centaurus	BA.2.75	BA.2.75, BA.2.75.1 BA.2.75.2, BA.2.75.4 BA.2.75.5, BA.2.75.6 BA.2.75.7, BL.1, BL.1.3, BL.2, BL.2.1 BL.3, BL.5, BM.1.1 BM.1.1.1, BM.1.1.3 BM.3, BM.4.1.1, BM.5 BM.6, BN.1, BN.1.1 BN.1.2, BN.1.3, BN.1.3.1, BN.1.3.3 BN.1.4, BN.1.5, BN.1.7 BN.2, BN.3.1, BN.5 BN.6, BR.1, BY.1 BY.1.1.1, CA.2, CA.3 CA.6, CA.7, CB.1 CH.1.1, CH.1.1.2, CH.2 CJ.1	BA.2.75, BA.2.75.1 BA.2.75.2, BA.2.75.3 BA.2.75.4, BA.2.75.5 BA.2.75.6, BA.2.75.7 BA.2.75.9, BL.1 BL.1.2, BL.1.3, BL.1.4, BL.2. BL.2.1 BL.3, BL.4, BM.1.1 BM.1.1.1, BM.1.1.3 BM.2, BM.4.1 BM.4.1.1, BN.1 BN.1.1, BN.1.2, BN.1.3, BN.1.3.1 BN.1.3.2, BN.1.3.3 BN.1.4, BN.1.5, BN.1.6, BN.1.9, BN.2, BN.3, BN.3.1 BN.4, BN.5, BN.6 BR.1, BR.1.2, BR.2.1 BR.3, BY.1, BY.1.1 BY.1.1.1, BY.1.2 CA.1, CA.2, CA.3 CA.6, CA.7, CB.1 CH.1.1, CH.2, CJ.1 CV.1	BA.2.75, BA.2.75.1 BA.2.75.2, BA.2.75.3 BA.2.75.4, BA.2.75.5 BA.2.75.6, BA.2.75.7 BA.2.75.8, BA.2.75.9 BA.2.75.10, BL.1, BL.1.3, BL.1.4, BL.2 BL.3, BL.6, BM.1.1 BM.1.1.1, BM.1.1.3 BM.1.1.4, BM.4.1 BM.4.1.1, BM.5, BN.1 BN.1.1, BN.1.1.1, BN.1.2, BN.1.2.1, BN.1.3, BN.1.3.1 BN.1.4, BN.1.5, BN.1.6, BN.1.9, BN.2 BN.3.1, BN.4, BN.5 BR.2.1, BR.5, BY.1 CA.3, CA.5, CA.6 CB.1, CH.1.1, CV.1	BA.2.75, BA.2.75.1 BA.2.75.2, BA.2.75.4, BL.1 BM.1, BM.1.1 BM.1.1.3, BN.1.1 BN.1.4, BY.1, CH.1.1	BA.2.75, BA.2.75.1 BA.2.75.2, BA.2.75.3 BA.2.75.4, BA.2.75.5 BA.2.75.6, BA.2.75.7 BA.2.75.10, BL.1, BL.1.1, BL.1.3, BL.1.4 BL.2, BL.3, BL.4, BL.5 BM.1, BM.1.1, BM.1.1.1, BM.1.1.2 BM.1.1.3, BM.2.3 BM.4.1, BM.4.1.1 BM.5, BN.1, BN.1.2 BN.1.2.1, BN.1.3, BN.1.3.1, BN.1.3.2, BN.1.4, BN.1.4.1, BN.1.5, BN.1.6, BN.1.7 BN.1.9, BN.2.1, BN.3 BN.3.1, BN.4, BN.5 BN.6, BR.1, BR.3, BR.5 BY.1, BY.1.2, BY.1.2.1 CA.1, CA.2, CA.3, CA.3.1, CA.5, CA.6 CA.7, CB.1, CH.1, CH.1.1, CH.1.1.2, CH.1.1.9, CJ.1, CV.1	BA.2.75, BA.2.75.1 BA.2.75.2, BA.2.75.3 BA.2.75.4, BA.2.75.5 BA.2.75.6, BA.2.75.7 BA.2.75.9, BA.2.75.10 BL.1, BL.1.3, BL.1.4, BL.2 BL.2.1, BL.3, BL.4, BL.6 BM.1, BM.1.1, BM.1.1.1 BM.1.1.3, BM.1.1.4, BM.2 BM.2.1, BM.3, BM.4, BM.4.1, BM.4.1.1, BM.5 BM.6, BN.1, BN.1.1, BN.1.1.1, BN.1.2, BN.1.3 BN.1.3.1, BN.1.3.2, BN.1.3.3, BN.1.4, BN.1.5 BN.1.5.1, BN.1.6, BN.1.7 BN.1.8, BN.1.9, BN.1.10 BN.2, BN.2.1, BN.3, BN.3.1, BN.4, BN.5, BN.6 BR.1, BR.1.2, BR.2.1, BR.3 BR.4, BR.5, BY.1, BY.1.1, BY.1.2, CA.1, CA.2, CA.3 CA.3.1, CA.5, CA.7, CB.1 CH.1, CH.1.1, CH.1.1.2, CH.2, CJ.1, CV.1, CV.2 DS.2	BA.2.75, BA.2.75.1, BA.2.75.2 BA.2.75.3, BA.2.75.4, BA.2.75.5, BA.2.75.6, BA.2.75.7, BA.2.75.8, BA.2.75.9, BA.2.75.10, BL.1 BL.1.1, BL.1.2, BL.1.3, BL.1.4 BL.2, BL.2.1, BL.3, BL.4, BL.5 BL.6, BM.1, BM.1.1, BM.1.1.1 BM.1.1.2, BM.1.1.3, BM.1.1.4 BM.1.1.5, BM.2, BM.2.1, BM.2.2, BM.2.3, BM.3, BM.4 BM.4.1, BM.4.1.1, BM.5, BM.6, BN.1, BN.1.1, BN.1.1.1 BN.1.2, BN.1.2.1, BN.1.3, BN.1.3.1, BN.1.3.2, BN.1.3.3 BN.1.3.4, BN.1.4, BN.1.4.1, BN.1.5, BN.1.5.1, BN.1.6, BN.1.7, BN.1.8, BN.1.9, BN.1.10, BN.2, BN.2.1, BN.3 BN.3.1, BN.4, BN.5, BN.6, BR.1, BR.1.1, BR.1.2, BR.2, BR.2.1, BR.3, BR.4, BR.5, BY.1, BY.1.1, BY.1.1.1, BY.1.2, BY.1.2.1, CA.1, CA.2 CA.3, CA.3.1, CA.4, CA.5, CA.6, CA.7, CB.1, CH.1, CH.1.1, CH.1.1.1, CH.1.1.2

									CH.1.1.3, CH.1.1.7, CH.1.1.9 CH.2, CH.3, CJ.1, CJ.1.1, CV.1 CV.2, DS.2
X					XA, XC, XF, XS, XAA, XAB, XAN, XAR, XBE, XBJ, XBB				XAA, XAB, XAN

Supplementary Materials S3. Dominant Sub-lineage in Max Share and Sample Size Table by Regions, and the Number of Sequences in Data

Yr-Week	Denmark			Germany			Korea			South Africa			UK			US			World		
	MxShare	Smpl Sz	Dom.	MxShare	Smpl Sz	Dom.	MxShare	Smpl Sz	Dom.	MxShare	Smpl Sz	Dom.	MxShare	Smpl Sz	Dom.	MxShare	Smpl Sz	Dom.	MxShare	Smpl Sz	Dom.
2020-33	0.6969	287	B.1.1	0.3151	73	B.1.1	0.7556	90	B.1.497	0.2387	155	B.1.1	0.3988	850	B.1.1	0.5035	2556	B.1	0.3059	5825	B.1
2020-34	0.4673	214	B.1.1	0.2642	53	B.1.1	0.9286	112	B.1.497	0.2603	73	B.1.1	0.3145	989	B.1.1	0.5698	2499	B.1	0.3097	5931	B.1
2020-35	0.4126	206	B.1.1	0.4423	52	B.1.1	0.9242	66	B.1.497	0.2985	67	B.1.1	0.2549	1526	B.1.177	0.5446	2868	B.1	0.3238	5964	B.1
2020-36	0.2567	635	B.1.1	0.5417	24	B.1.1	0.9250	40	B.1.497	0.2785	158	B.1.1	0.2791	1501	B.1.177	0.4870	2425	B.1	0.2927	5480	B.1
2020-37	0.3389	239	B.1.1	0.2000	50	AY.43	1.0000	20	B.1.497	0.2749	211	B.1.1	0.3848	2279	B.1.177	0.5198	2545	B.1	0.2791	5948	B.1
2020-38	0.2301	704	B.1.177.21	0.3103	87	B.1.177	0.8261	23	B.1.497	0.3154	130	B.1.351	0.4458	2723	B.1.177	0.4569	3248	B.1	0.2551	7045	B.1.177
2020-39	0.2027	804	B.1.1	0.4206	126	B.1.1	0.8000	30	B.1.497	0.3400	100	B.1.1	0.4669	4474	B.1.177	0.4366	3658	B.1	0.3190	8683	B.1.177
2020-40	0.1908	393	B.1.160	0.3565	115	B.1.1	0.6154	26	B.1.497	0.2951	122	B.1.351	0.4823	3450	B.1.177	0.3906	3308	B.1.2	0.2977	7925	B.1.177
2020-41	0.2219	320	B.1.160	0.2903	124	B.1.1	0.8519	27	B.1.497	0.2337	184	B.1.1	0.5136	5173	B.1.177	0.4075	4820	B.1.2	0.3419	10797	B.1.177
2020-42	0.2346	942	B.1.160	0.1750	120	B.1.177	0.8333	24	B.1.497	0.3616	177	B.1.351	0.5652	5147	B.1.177	0.4257	3968	B.1.2	0.3726	10726	B.1.177
2020-43	0.2472	267	B.1.160	0.1930	114	B.1.1	0.9231	39	B.1.497	0.4021	189	B.1.351	0.5633	7135	B.1.177	0.4850	4639	B.1.2	0.3795	13032	B.1.177
2020-44	0.1730	1243	B.1.160	0.2529	174	B.1.1	0.9302	43	B.1.497	0.4518	228	B.1.351	0.6073	5943	B.1.177	0.5193	5775	B.1.2	0.3380	13949	B.1.177
2020-45	0.1597	1246	B.1.160	0.2865	171	B.1.1	0.9630	27	B.1.497	0.5984	249	B.1.351	0.6179	8010	B.1.177	0.5670	8250	B.1.2	0.3243	18183	B.1.177
2020-46	0.1788	1074	B.1.177.21	0.3204	181	B.1.1	0.9851	67	B.1.497	0.6899	158	B.1.351	0.5841	8587	B.1.177	0.5750	7452	B.1.2	0.3354	17942	B.1.177
2020-47	0.2285	1619	B.1.177.21	0.2715	221	B.1.1	0.9588	97	B.1.497	0.7249	269	B.1.351	0.5781	5784	B.1.177	0.6160	6810	B.1.2	0.2822	14948	B.1.2
2020-48	0.2684	2001	B.1.177.21	0.3516	219	B.1.1	0.8974	78	B.1.497	0.8402	244	B.1.351	0.5669	3572	B.1.177	0.6457	5213	B.1.2	0.2768	12264	B.1.2
2020-49	0.3251	2658	B.1.177.21	0.2321	280	B.1.177	0.8947	95	B.1.497	0.9183	257	B.1.351	0.5625	3264	B.1.177	0.6008	8329	B.1.2	0.3198	15811	B.1.2
2020-50	0.3309	3968	B.1.177.21	0.1720	372	B.1.258	0.9143	70	B.1.497	0.9203	251	B.1.351	0.4330	8236	B.1.177	0.5047	10474	B.1.2	0.2361	23552	B.1.2
2020-51	0.3200	4722	B.1.177.21	0.2662	308	B.1.221	0.8448	116	B.1.497	0.9691	356	B.1.351	0.5174	10829	B.1.1.7	0.4995	9790	B.1.2	0.2162	26984	B.1.1.7
2020-52	0.3020	3507	B.1.177.21	0.1452	303	B.1.221	0.8077	104	B.1.497	0.9418	447	B.1.351	0.5884	7070	B.1.1.7	0.5654	7727	B.1.2	0.2251	21832	B.1.1.7
2020-53	0.2735	3763	B.1.177.21	0.2123	212	B.1.258	0.6860	86	B.1.497	0.9682	503	B.1.351	0.7627	10283	B.1.1.7	0.5538	10291	B.1.2	0.3131	29450	B.1.1.7
2021-1	0.2624	3940	B.1.177.21	0.2299	361	B.1.177	0.6857	70	B.1.497	0.9181	708	B.1.351	0.8027	11980	B.1.1.7	0.5299	16866	B.1.2	0.2992	40683	B.1.1.7
2021-2	0.2682	4109	B.1.177.21	0.1546	854	B.1.258	0.5536	56	B.1.497	0.9413	392	B.1.351	0.8441	15300	B.1.1.7	0.5008	17943	B.1.2	0.3487	46637	B.1.1.7
2021-3	0.2415	3453	B.1.177.21	0.1471	1618	B.1.1.7	0.7204	93	B.1.497	0.9833	239	B.1.351	0.8940	14423	B.1.1.7	0.5150	15832	B.1.2	0.3914	45189	B.1.1.7
2021-4	0.2177	2389	B.1.177.21	0.2061	2445	B.1.1.7	0.6818	110	B.1.497	0.9725	218	B.1.351	0.9401	20608	B.1.1.7	0.4580	15411	B.1.2	0.5061	53566	B.1.1.7
2021-5	0.3212	2027	B.1.1.7	0.2788	2841	B.1.1.7	0.5905	105	B.1.497	0.9720	214	B.1.351	0.9442	12128	B.1.1.7	0.3816	17974	B.1.2	0.4569	50348	B.1.1.7
2021-6	0.5231	1776	B.1.1.7	0.4735	3605	B.1.1.7	0.4747	158	B.1.497	0.9701	167	B.1.351	0.9686	15612	B.1.1.7	0.3955	19025	B.1.2	0.5180	56954	B.1.1.7
2021-7	0.7000	2270	B.1.1.7	0.5682	4477	B.1.1.7	0.4247	259	A.18	0.9882	169	B.1.351	0.9777	16879	B.1.1.7	0.3765	15664	B.1.2	0.5981	57777	B.1.1.7
2021-8	0.7864	2575	B.1.1.7	0.5769	4945	B.1.1.7	0.5281	231	A.18	0.9677	93	B.1.351	0.9845	16104	B.1.1.7	0.3267	17684	B.1.2	0.6384	63507	B.1.1.7
2021-9	0.8542	2648	B.1.1.7	0.6782	5501	B.1.1.7	0.4510	306	A.18	0.9636	165	B.1.351	0.9899	13818	B.1.1.7	0.2746	20217	B.1.2	0.6573	70054	B.1.1.7
2021-10	0.9092	2920	B.1.1.7	0.7738	6460	B.1.1.7	0.4702	285	B.1.497	0.9470	283	B.1.351	0.9905	18185	B.1.1.7	0.3350	22712	B.1.1.7	0.7125	83106	B.1.1.7
2021-11	0.8976	3300	B.1.1.7	0.8420	7534	B.1.1.7	0.4906	267	A.18	0.8106	227	B.1.351	0.9967	16660	B.1.1.7	0.4281	27126	B.1.1.7	0.7402	89448	B.1.1.7
2021-12	0.9197	3650	B.1.1.7	0.8709	7963	B.1.1.7	0.4594	320	A.18	0.8883	179	B.1.351	0.9975	17012	B.1.1.7	0.5054	30633	B.1.1.7	0.7597	93535	B.1.1.7
2021-13	0.9362	3388	B.1.1.7	0.8886	8288	B.1.1.7	0.5475	495	A.18	0.8383	167	B.1.351	0.9966	12450	B.1.1.7	0.5809	35857	B.1.1.7	0.7478	93186	B.1.1.7
2021-14	0.9584	3315	B.1.1.7	0.9086	8373	B.1.1.7	0.3599	464	A.18	0.9134	127	B.1.351	0.9890	8126	B.1.1.7	0.6292	39987	B.1.1.7	0.7434	94155	B.1.1.7
2021-15	0.9714	3569	B.1.1.7	0.9173	11061	B.1.1.7	0.2806	392	A.18	0.8500	160	B.1.351	0.9744	7576	B.1.1.7	0.6458	38503	B.1.1.7	0.7701	98211	B.1.1.7
2021-16	0.9531	3474	B.1.1.7	0.9348	10208	B.1.1.7	0.2722	529	B.1.1.7	0.8816	245	B.1.351	0.9407	7221	B.1.1.7	0.6668	33548	B.1.1.7	0.7806	94144	B.1.1.7
2021-17	0.9643	3870	B.1.1.7	0.9164	8903	B.1.1.7	0.3525	590	B.1.1.7	0.8221	163	B.1.351	0.8831	7049	B.1.1.7	0.6826	33627	B.1.1.7	0.7645	95029	B.1.1.7
2021-18	0.9769	4634	B.1.1.7	0.9065	8891	B.1.1.7	0.2308	455	B.1.619.1	0.7862	318	B.1.351	0.7637	7142	B.1.1.7	0.6882	28661	B.1.1.7	0.7668	86838	B.1.1.7
2021-19	0.9828	4706	B.1.1.7	0.8895	6245	B.1.1.7	0.3416	281	B.1.1.7	0.6887	257	B.1.351	0.5972	7199	B.1.1.7	0.6724	21846	B.1.1.7	0.7472	74973	B.1.1.7
2021-20	0.9916	4643	B.1.1.7	0.8984	5215	B.1.1.7	0.3640	261	B.1.619.1	0.6921	328	B.1.351	0.4198	9155	AY.4	0.6662	17152	B.1.1.7	0.6930	68382	B.1.1.7
2021-21	0.9882	4666	B.1.1.7	0.8740	4127	B.1.1.7	0.4951	410	B.1.619.1	0.5928	388	B.1.351	0.5399	14168	AY.4	0.6263	11787	B.1.1.7	0.6052	60473	B.1.1.7
2021-22	0.9801	3823	B.1.1.7	0.8245	2957	B.1.1.7	0.6136	396	B.1.619.1	0.4671	623	B.1.351	0.6203	21737	AY.4	0.5480	9226	B.1.1.7	0.4592	61290	B.1.1.7

Yr-Week	Denmark			Germany			Korea			South Africa			UK			US			World		
	MxShare	Smpl Sz	Dom.	MxShare	Smpl Sz	Dom.	MxShare	Smpl Sz	Dom.	MxShare	Smpl Sz	Dom.	MxShare	Smpl Sz	Dom.	MxShare	Smpl Sz	Dom.	MxShare	Smpl Sz	Dom.
2021-23	0.9642	1983	B.1.1.7	0.7331	2072	B.1.1.7	0.6270	370	B.1.619.1	0.3350	591	B.1.351	0.6451	14850	AY.4	0.4802	10022	B.1.1.7	0.4438	53650	B.1.1.7
2021-24	0.9109	1066	B.1.1.7	0.5041	1339	B.1.1.7	0.4949	390	B.1.619.1	0.2675	830	AY.45	0.7602	18259	AY.4	0.3839	9828	B.1.1.7	0.3134	51173	B.1.1.7
2021-25	0.6684	944	B.1.1.7	0.3738	971	B.1.1.7	0.3752	773	B.1.619.1	0.3569	1087	AY.45	0.7541	25778	AY.4	0.2441	11418	B.1.1.7	0.3289	60393	AY.4
2021-26	0.3062	2077	B.1.1.7	0.3075	1060	AY.122	0.2992	909	B.1.619.1	0.4261	1204	AY.45	0.7432	31918	AY.4	0.1954	13822	AY.44	0.3431	72108	AY.4
2021-27	0.2567	2809	AY.7.1	0.2858	1081	AY.122	0.2954	1046	B.1.619.1	0.5427	1229	AY.45	0.7427	26765	AY.4	0.1927	22974	AY.44	0.2591	82937	AY.4
2021-28	0.2981	5586	AY.7.1	0.2221	1378	AY.122	0.2677	1072	AY.69	0.5221	906	AY.45	0.7249	24132	AY.4	0.1975	33310	AY.44	0.1997	101013	AY.4
2021-29	0.2477	4219	AY.7.1	0.1954	2221	AY.43	0.2945	883	B.1.617.2	0.5354	861	AY.45	0.7442	34700	AY.4	0.1902	50934	AY.44	0.2086	139840	AY.4
2021-30	0.2502	5012	AY.43	0.1937	2266	AY.122	0.4217	894	AY.69	0.3834	999	AY.45	0.7380	32033	AY.4	0.1834	68782	AY.44	0.1718	155859	AY.4
2021-31	0.2136	4995	AY.43	0.2019	2898	AY.122	0.5423	603	AY.69	0.5076	851	AY.45	0.7527	32121	AY.4	0.1728	77298	AY.44	0.1586	173429	AY.4
2021-32	0.2163	5299	AY.43	0.2138	4682	AY.122	0.5660	523	AY.69	0.4371	684	AY.45	0.7506	29687	AY.4	0.1827	66851	AY.44	0.1533	167644	AY.4
2021-33	0.1773	4918	AY.43	0.2000	6731	AY.122	0.5963	706	AY.69	0.4874	792	AY.45	0.7182	35439	AY.4	0.1760	65025	AY.44	0.1687	193900	B.1.617.2
2021-34	0.1570	4726	AY.122	0.1821	8389	AY.122	0.4882	932	AY.69	0.5088	678	AY.45	0.7323	38560	AY.4	0.1760	66665	AY.44	0.1860	173360	AY.4
2021-35	0.1324	3202	AY.122	0.1679	8801	AY.122	0.6200	1100	AY.69	0.5014	692	AY.45	0.7313	39691	AY.4	0.1756	72145	AY.103	0.1902	174305	AY.4
2021-36	0.1395	2488	AY.122	0.1954	9802	AY.43	0.6352	1006	AY.69	0.4324	562	AY.45	0.7280	39310	AY.4	0.1851	72232	AY.103	0.1965	167519	AY.4
2021-37	0.1414	1775	AY.122	0.1989	8387	AY.43	0.5375	733	AY.69	0.4658	483	AY.45	0.7095	39177	AY.4	0.1915	65421	AY.103	0.2131	148935	AY.4
2021-38	0.1475	2129	AY.122	0.1915	7793	AY.43	0.5376	599	AY.69	0.3878	361	AY.45	0.6842	37576	AY.4	0.1962	57177	AY.103	0.2169	134587	AY.4
2021-39	0.1593	2517	AY.4	0.1950	6646	AY.43	0.6011	702	AY.69	0.4615	208	AY.45	0.6857	37610	AY.4	0.2046	57534	AY.103	0.2155	137190	AY.4
2021-40	0.2028	3245	AY.4	0.2007	6167	AY.43	0.6068	679	AY.69	0.4195	298	AY.45	0.6714	40384	AY.4	0.2129	58794	AY.103	0.2247	140224	AY.4
2021-41	0.2180	4041	AY.4	0.1944	6476	AY.43	0.6594	737	AY.69	0.4100	239	AY.45	0.6435	36680	AY.4	0.2185	52296	AY.103	0.2196	130319	AY.4
2021-42	0.2237	7058	AY.4	0.2292	7443	AY.43	0.5793	1179	AY.69	0.4476	143	AY.45	0.6331	42025	AY.4	0.2221	53303	AY.103	0.2253	145406	AY.4
2021-43	0.2051	7952	AY.4	0.2365	7071	AY.43	0.4466	1021	AY.69	0.4949	99	AY.45	0.6298	43676	AY.4	0.2301	54847	AY.103	0.2282	153060	AY.4
2021-44	0.2034	10600	AY.4	0.2367	7612	AY.43	0.4954	1088	AY.69	0.3585	53	AY.45	0.6022	44469	AY.4	0.2430	59505	AY.103	0.2110	162750	AY.4
2021-45	0.1989	10841	AY.43	0.2390	9086	AY.43	0.4842	979	AY.69	0.3871	62	BA.1*	0.5783	52191	AY.4	0.2513	62156	AY.103	0.2179	172316	AY.4
2021-46	0.1943	11076	AY.43	0.2522	11040	AY.43	0.4258	890	AY.69	0.8401	319	BA.1*	0.5514	52695	AY.4	0.2572	59218	AY.103	0.2092	176048	AY.4
2021-47	0.1769	11862	AY.4	0.2430	10731	AY.43	0.4195	975	AY.69	0.9244	754	BA.1*	0.5164	57494	AY.4	0.2630	47922	AY.103	0.2219	168435	AY.4
2021-48	0.1805	16813	AY.4	0.2475	11328	AY.43	0.4880	996	AY.69	0.9620	1581	BA.1*	0.4726	62384	AY.4	0.2595	67513	AY.103	0.1884	210407	AY.4
2021-49	0.1694	12589	AY.4	0.2346	12185	AY.43	0.4859	708	AY.69	0.9465	1478	BA.1*	0.3856	64175	AY.4	0.2465	62377	AY.103	0.1631	204276	AY.4
2021-50	0.4642	6849	BA.1*	0.2165	12625	AY.43	0.5046	989	AY.69	0.9326	1217	BA.1*	0.6216	70320	BA.1*	0.4192	75158	BA.1*	0.4630	226469	BA.1*
2021-51	0.6432	4921	BA.1*	0.2906	11398	BA.1*	0.4284	803	AY.122	0.9105	1050	BA.1*	0.8490	51252	BA.1*	0.7749	86932	BA.1*	0.7080	220578	BA.1*
2021-52	0.7231	10556	BA.1*	0.5926	12017	BA.1*	0.3515	697	AY.122	0.8107	655	BA.1*	0.9540	72147	BA.1*	0.9029	108835	BA.1*	0.8357	288511	BA.1*
2022-1	0.6660	9018	BA.1*	0.7226	12529	BA.1*	0.4710	1189	BA.1*	0.7214	840	BA.1*	0.9754	76480	BA.1*	0.9573	122211	BA.1*	0.8912	320144	BA.1*
2022-2	0.5372	13128	BA.2*	0.8066	11941	BA.1*	0.7592	789	BA.1*	0.6204	656	BA.1*	0.9761	66049	BA.1*	0.9789	109203	BA.1*	0.9043	296495	BA.1*
2022-3	0.7051	16050	BA.2*	0.8536	13872	BA.1*	0.8148	891	BA.1*	0.5556	711	BA.2*	0.9592	71916	BA.1*	0.9858	103186	BA.1*	0.8891	301830	BA.1*
2022-4	0.8162	13928	BA.2*	0.8436	15093	BA.1*	0.8392	597	BA.1*	0.6481	503	BA.2*	0.9208	64272	BA.1*	0.9856	89329	BA.1*	0.8727	264072	BA.1*
2022-5	0.8901	11668	BA.2*	0.7948	19823	BA.1*	0.8359	957	BA.1*	0.7305	282	BA.2*	0.8436	75454	BA.1*	0.9837	80656	BA.1*	0.8185	273253	BA.1*
2022-6	0.9328	13421	BA.2*	0.7213	18807	BA.1*	0.7964	953	BA.1*	0.8557	291	BA.2*	0.7466	73105	BA.1*	0.9738	72793	BA.1*	0.7593	251638	BA.1*
2022-7	0.9596	14572	BA.2*	0.5824	21757	BA.1*	0.7076	1149	BA.1*	0.9088	351	BA.2*	0.5824	73633	BA.1*	0.9513	50151	BA.1*	0.6452	227302	BA.1*
2022-8	0.9731	14135	BA.2*	0.5469	17895	BA.2*	0.5908	1063	BA.1*	0.8930	327	BA.2*	0.5707	57745	BA.2*	0.9054	34775	BA.1*	0.5311	187275	BA.1*
2022-9	0.9795	13119	BA.2*	0.6699	20926	BA.2*	0.4804	1299	BA.1*	0.9161	286	BA.2*	0.7497	62445	BA.2*	0.8233	23073	BA.1*	0.6108	175251	BA.2*
2022-10	0.9857	13510	BA.2*	0.7492	20998	BA.2*	0.4030	1201	BA.2*	0.8140	328	BA.2*	0.8476	74971	BA.2*	0.6700	18257	BA.1*	0.7284	185265	BA.2*
2022-11	0.9895	13276	BA.2*	0.8323	20860	BA.2*	0.3927	899	XAA	0.8095	315	BA.2*	0.9111	74490	BA.2*	0.5033	17986	BA.1*	0.8110	185156	BA.2*
2022-12	0.9898	12493	BA.2*	0.8888	15754	BA.2*	0.4478	1505	BA.2*	0.6777	363	BA.2*	0.9501	68190	BA.2*	0.6835	20349	BA.2*	0.8664	178621	BA.2*
2022-13	0.9932	12636	BA.2*	0.9233	14268	BA.2*	0.5355	1942	BA.2*	0.5040	375	BA.2*	0.9639	29452	BA.2*	0.8203	22710	BA.2*	0.8890	134860	BA.2*
2022-14	0.9961	11851	BA.2*	0.9416	14325	BA.2*	0.5582	1410	BA.2*	0.4403	511	BA.2*	0.9755	19979	BA.2*	0.9003	30561	BA.2*	0.9080	129489	BA.2*
2022-15	0.9965	8836	BA.2*	0.9693	14885	BA.2*	0.5037	1773	BA.2*	0.4660	397	BA.4*	0.9866	20020	BA.2*	0.9466	32189	BA.2*	0.9331	123197	BA.2*
2022-16	0.9974	7653	BA.2*	0.9762	13088	BA.2*	0.5283	1908	BA.2*	0.5777	637	BA.4*	0.9904	17946	BA.2*	0.9689	36298	BA.2*	0.9430	123511	BA.2*

Yr-Week	Denmark			Germany			Korea			South Africa			UK			US			World		
	MxShare	Smpl Sz	Dom.	MxShare	Smpl Sz	Dom.	MxShare	Smpl Sz	Dom.	MxShare	Smpl Sz	Dom.	MxShare	Smpl Sz	Dom.	MxShare	Smpl Sz	Dom.	MxShare	Smpl Sz	Dom.
2022-17	0.9922	4715	BA.2*	0.9786	12372	BA.2*	0.5817	1891	BA.2*	0.6820	915	BA.4*	0.9876	12707	BA.2*	0.9806	34720	BA.2*	0.9405	110026	BA.2*
2022-18	0.9874	4056	BA.2*	0.9746	10543	BA.2*	0.5357	1471	BA.2*	0.6768	885	BA.4*	0.9759	6979	BA.2*	0.9833	40745	BA.2*	0.9394	107272	BA.2*
2022-19	0.9768	3406	BA.2*	0.9507	9504	BA.2*	0.6016	1702	BA.2*	0.6271	472	BA.4*	0.9503	5730	BA.2*	0.9722	45505	BA.2*	0.9283	109290	BA.2*
2022-20	0.9418	2888	BA.2*	0.9225	8345	BA.2*	0.6649	1513	BA.2*	0.5946	661	BA.4*	0.8884	4246	BA.2*	0.9497	44497	BA.2*	0.8937	99718	BA.2*
2022-21	0.8188	2202	BA.2*	0.8286	7040	BA.2*	0.7665	1589	BA.2*	0.6057	563	BA.4*	0.7775	3487	BA.2*	0.9068	44825	BA.2*	0.8429	94525	BA.2*
2022-22	0.6433	2299	BA.2*	0.6332	7628	BA.2*	0.6288	1568	BA.2*	0.5786	458	BA.4*	0.5893	4171	BA.2*	0.8289	41850	BA.2*	0.7309	97650	BA.2*
2022-23	0.4696	3584	BA.5*	0.5152	8079	BA.5*	0.5574	1882	BA.2*	0.5313	320	BA.4*	0.4097	7191	BA.5*	0.7209	43373	BA.2*	0.6051	109172	BA.2*
2022-24	0.6260	5714	BA.5*	0.6453	11941	BA.5*	0.4483	1305	BA.2*	0.5650	200	BA.4*	0.5381	8869	BA.5*	0.5737	37777	BA.2*	0.4482	111613	BA.5*
2022-25	0.7328	4503	BA.5*	0.7594	14773	BA.5*	0.3121	1442	BA.2*	0.5193	181	BA.4*	0.6548	12403	BA.5*	0.4439	37703	BA.5*	0.5478	123286	BA.5*
2022-26	0.8239	4111	BA.5*	0.8291	12448	BA.5*	0.2917	1683	BA.5*	0.5540	139	BA.5*	0.7205	14633	BA.5*	0.5757	41365	BA.5*	0.6361	133628	BA.5*
2022-27	0.8545	3932	BA.5*	0.8755	8313	BA.5*	0.4105	1593	BA.5*	0.7023	131	BA.5*	0.7912	13628	BA.5*	0.6826	44414	BA.5*	0.7089	136713	BA.5*
2022-28	0.9032	3947	BA.5*	0.9017	7946	BA.5*	0.5196	1405	BA.5*	0.6164	146	BA.5*	0.8195	10515	BA.5*	0.7535	46610	BA.5*	0.7666	136634	BA.5*
2022-29	0.9220	4332	BA.5*	0.9255	8122	BA.5*	0.4605	734	BA.5*	0.7544	114	BA.5*	0.8512	9521	BA.5*	0.8049	41528	BA.5*	0.8094	125606	BA.5*
2022-30	0.9203	3826	BA.5*	0.9433	6242	BA.5*	0.5934	2415	BA.5*	0.6563	64	BA.5*	0.8705	7786	BA.5*	0.8372	38439	BA.5*	0.8232	107522	BA.5*
2022-31	0.9457	3207	BA.5*	0.9530	5105	BA.5*	0.5353	2038	BA.5*	0.6795	78	BA.5*	0.8742	6016	BA.5*	0.8505	35158	BA.5*	0.8320	98971	BA.5*
2022-32	0.9404	3606	BA.5*	0.9624	4920	BA.5*	0.6237	1488	BA.5*	0.7424	66	BA.5*	0.8950	4886	BA.5*	0.8683	32141	BA.5*	0.8479	87222	BA.5*
2022-33	0.9401	2619	BA.5*	0.9642	4577	BA.5*	0.8003	2794	BA.5*	0.6892	74	BA.5*	0.9039	3818	BA.5*	0.8763	32192	BA.5*	0.8546	83757	BA.5*
2022-34	0.9429	2627	BA.5*	0.9626	4551	BA.5*	0.7066	2301	BA.5*	0.6567	67	BA.5*	0.8971	3002	BA.5*	0.8726	30905	BA.5*	0.8597	77964	BA.5*
2022-35	0.9425	3060	BA.5*	0.9566	4635	BA.5*	0.8213	1774	BA.5*	0.8529	68	BA.5*	0.8952	3091	BA.5*	0.8673	27960	BA.5*	0.8490	72716	BA.5*
2022-36	0.9252	3447	BA.5*	0.9599	6186	BA.5*	0.7984	1111	BA.5*	0.7540	126	BA.5*	0.8923	2794	BA.5*	0.8637	23324	BA.5*	0.8408	65877	BA.5*
2022-37	0.9288	3510	BA.5*	0.9544	6232	BA.5*	0.7949	2331	BA.5*	0.8065	186	BA.5*	0.8906	3172	BA.5*	0.8598	25655	BA.5*	0.8458	68046	BA.5*
2022-38	0.9404	3761	BA.5*	0.9598	4625	BA.5*	0.7404	1849	BA.5*	0.6831	183	BA.5*	0.8813	4686	BA.5*	0.8627	23036	BA.5*	0.8345	62891	BA.5*
2022-39	0.9382	3592	BA.5*	0.9621	4729	BA.5*	0.7258	1816	BA.5*	0.9000	160	BA.5*	0.8851	5337	BA.5*	0.8626	19433	BA.5*	0.8439	54640	BA.5*
2022-40	0.9494	4011	BA.5*	0.9613	5885	BA.5*	0.8255	1799	BA.5*	0.9000	210	BA.5*	0.8892	7186	BA.5*	0.8726	15877	BA.5*	0.8511	51370	BA.5*

DK: Denmark; DE: Germany; KR: Korea; ZA: South Africa; UK: United Kingdom; US: United States

2020-01-06 to 2022-10-31	DK	DE	KR	ZA	UK	US	World
Total Sequences	596656	835858	100564	48424	2856402	4314779	13757463
Collected Sequences	578900	800675	99839	47797	2768358	4126817	12354651
Used Sequences	566354	779170	92315	46618	2751551	4074523	12296756
Proportion we used	0.949214	0.93218	0.917973	0.962704	0.963293	0.944318	0.893824392

Source: covSPECTRUM, 17 Feb.2023, https://cov-spectrum.org/explore/World/AllSamples/AllTimes/variants?&

Supplementary Materials S4:

1. Estimating the probability of the phase-shift state

Suppose we have the inferred probability in the previous period, $\Pr(S_{t-1} = j|\boldsymbol{\Omega}_{t-1}, \boldsymbol{\Theta})$. Using Bayes rule, we can write the conditional pdf as

$$f(y_t|\boldsymbol{\Omega}_{t-1}, \boldsymbol{\Theta}) = \sum_{i=0}^{1} \sum_{j=0}^{1} f(y_t|S_t = j, \boldsymbol{\Omega}_{t-1}) \Pr(S_{t-1} = i, S_t = j|\boldsymbol{\Omega}_{t-1}, \boldsymbol{\Theta}) \quad (4)$$

$$= \sum_{i=0}^{1} \sum_{j=0}^{1} f(y_t|S_t = j, \boldsymbol{\Omega}_{t-1}) \Pr(S_t = j|S_{t-1} = i, \boldsymbol{\Theta}) \Pr(S_{t-1} = i|\boldsymbol{\Omega}_{t-1}, \boldsymbol{\Theta}), \quad (5)$$

Where $\Pr(S_t = j|S_{t-1} = i, \boldsymbol{\Theta}) = p_{ij}$ and $f(y_t|S_t = j, \boldsymbol{\Omega}_{t-1}, \boldsymbol{\Theta})$ is the cpdf of $N\left(\mu + \phi y_{t-1} - \delta\epsilon_{t-1}, \sigma_j^2\right)$ for j=0,1,[1]

$$f(y_t|S_t = j, \boldsymbol{\Omega}_{t-1}, \boldsymbol{\Theta}) = \frac{1}{\sigma_j\sqrt{2\pi}} exp\left(-\frac{(y_t - \mu - \phi y_{t-1} - \delta\epsilon_{t-1})^2}{2\sigma_j^2}\right).$$

Then we have the updated probability of S_t as

$$\Pr(S_t = j|\boldsymbol{\Omega}_t, \boldsymbol{\Theta}) = \sum_{i=0}^{1} \frac{f(y_t|S_t = j, \boldsymbol{\Omega}_{t-1}, \boldsymbol{\Theta}) \Pr(S_t = j, S_{t-1} = i|\boldsymbol{\Omega}_{t-1}, \boldsymbol{\Theta})}{f(y_t|\boldsymbol{\Omega}_{t-1}, \boldsymbol{\Theta})}. \quad (6)$$

Starting from $t = 1$, we can compute the inferred probability and the conditional pdf for $t = 1, \cdots, T$ by reassigning t to $t - 1$ after calculating the updated probability in (6) and starting the next iteration from (4). Note that as a byproduct of Newton-Raphson algorithm[2], we obtain the value of the log likelihood function from (5), which is the object to maximize in the MLE. After estimating the parameters $\widehat{\boldsymbol{\Theta}}_{ML}$, we use the filter in equations (4) to (6) again after plugging in $\widehat{\boldsymbol{\Theta}}_{ML}$ in the place of $\boldsymbol{\Theta}$ to compute the inferred probability $\Pr\left(S_t = 1\middle|\boldsymbol{\Omega}_t, \widehat{\boldsymbol{\Theta}}_{ML}\right)$ and the predicted probability for $\Pr\left(S_t = 1\middle|\boldsymbol{\Omega}_{t-1}, \widehat{\boldsymbol{\Theta}}_{ML}\right)$ $t = 1,2, \cdots, T$. Using the inferred and predicted probabilities, the smoothed probability $\Pr\left(S_t = 1\middle|\boldsymbol{\Omega}_T, \widehat{\boldsymbol{\Theta}}_{ML}\right)$ is obtained with Kim's smoothing algorithm (Kim, 1994) as below[3]:

---

[1] We use ARMA (1,1) setup based on the statistical significance of the model coefficients.

[2] Based on Bayesian information criteria, we chose ARMA (1,1) and the set of parameters is $\Theta = \{\mu, \phi_1, \delta_1, \sigma_0^2, \sigma_1^2, p_{00}, p_{11}\}$. The optimization algorithm updates the values of parameters from Θ_n to Θ_{n+1} using the value of gradient of the likelihood function evaluated at Θ_n until the minimum distance Θ_n and Θ_{n+1} becomes smaller than a presumed value of the threshold.

[3] The inferred probability $\Pr\left(S_t = 1\middle|\boldsymbol{\Omega}_t, \widehat{\boldsymbol{\Theta}}_{ML}\right)$ is estimated with the information up to period t for each $t = 1,2, \cdots, T$, while the smoothed probability is using the full sample information up to period T. For the

$$\Pr\left(S_t = 1 \middle| \mathbf{\Omega}_T, \widehat{\mathbf{\Theta}}_{ML}\right) = \sum_{k=0}^{1} \frac{\Pr\left(S_{t+1} = k \middle| \mathbf{\Omega}_T, \widehat{\mathbf{\Theta}}_{ML}\right) \Pr\left(S_t = j \middle| \mathbf{\Omega}_t, \widehat{\mathbf{\Theta}}_{ML}\right) p_{jk}}{\Pr\left(S_{t+1} = k \middle| \mathbf{\Omega}_t, \widehat{\mathbf{\Theta}}_{ML}\right)} \tag{7}$$

2. Forecasting probability of the phase shift

The out-of-sample probability of S_{t+h} for $h > 0$ is recursively computed using following relationship:

$$\begin{aligned}\Pr\left(S_{T+h} = j \middle| \mathbf{\Omega}_T, \widehat{\mathbf{\Theta}}_{ML}\right) &= \sum_{i=0}^{1} \Pr\left(S_{T+h} = j \middle| S_{T+h-1} = i, \widehat{\mathbf{\Theta}}_{ML}\right) \Pr\left(S_{T+h-1} = i \middle| \mathbf{\Omega}_T, \widehat{\mathbf{\Theta}}_{ML}\right) \\ &= \sum_{i=0}^{1} p_{ij} \Pr\left(S_{T+h-1} = j \middle| \mathbf{\Omega}_T, \widehat{\mathbf{\Theta}}_{ML}\right)\end{aligned} \tag{8}$$

One can interpret $\Pr\left(S_{T+h} = j \middle| \mathbf{\Omega}_T, \widehat{\mathbf{\Theta}}_{ML}\right)$ as the probability of observing phase-shift regime in $T + h$th week given the data of the values of the largest share among the sublineage groups up to Tth week.

proof of equation (7), the readers may refer to Kim (1994) or Kim and Nelson (1999)

Supplementary Materials S5. Unit Root Test

	ADF statistics	P-value
Denmark	-1.729	0.414
Germany	-1.160	0.690
Korea	-2.510	0.116
South Africa	-2.175	0.217
United Kingdom	-2.192	0.210
United States	-2.112	0.240
World	-2.260	0.187

Note: ADF statistics refers to the Augmented Dickey-Fuller test statistics, where lag length of each test equation is chosen based on Schwarz information criterion. The sample period is from 8/10/2020 to 10/3/2022 as in the sample period for the model estimation. The 1%, 5%, and 10% critical values are -3.489, -2.887, and -2.581, respectively.

Supplementary Materials S6. Normality/Heteroscedasticity test

Jarque-Bera normality test

H0: regression errors are normally distributed

Equation Sample: 8/10/2020 to 10/03/2022

	Skewness	Kurtosis	JB statistics	P-value
Denmark	-0.550	7.772	112.930	0.000
Germany	-0.753	7.845	121.204	0.000
Korea	0.373	3.095	2.663	0.264
South Africa	0.981	6.504	75.936	0.000
United Kingdom	0.975	6.336	70.326	0.000
United States	1.394	9.062	209.594	0.000
World	11.120	7.757	130.136	0.000

Quandt-Andrews break test (Korea)

H0: Mean of squared error is time constant

Equation Sample: 6/22/2020 to 10/31/2022

Test sample: 8/10/2020 9/19/2022

Statistics	Value	P-value
Maximum LR F-statistics	16.633	0.002
Expected LR F-statistics	4.394	0.001
Average LR F-statistics	3.391	0.023

Note: LR F-statistics refers to log-likelihood F-statistics. Probabilities are calculated using Hansen's method [1]. The equation sample was extended to match the first period of 5% trimmed data to the first period of the model estimation period.

Supplementary Materials S7. Max Share and the probability of phase shift depending on countries.

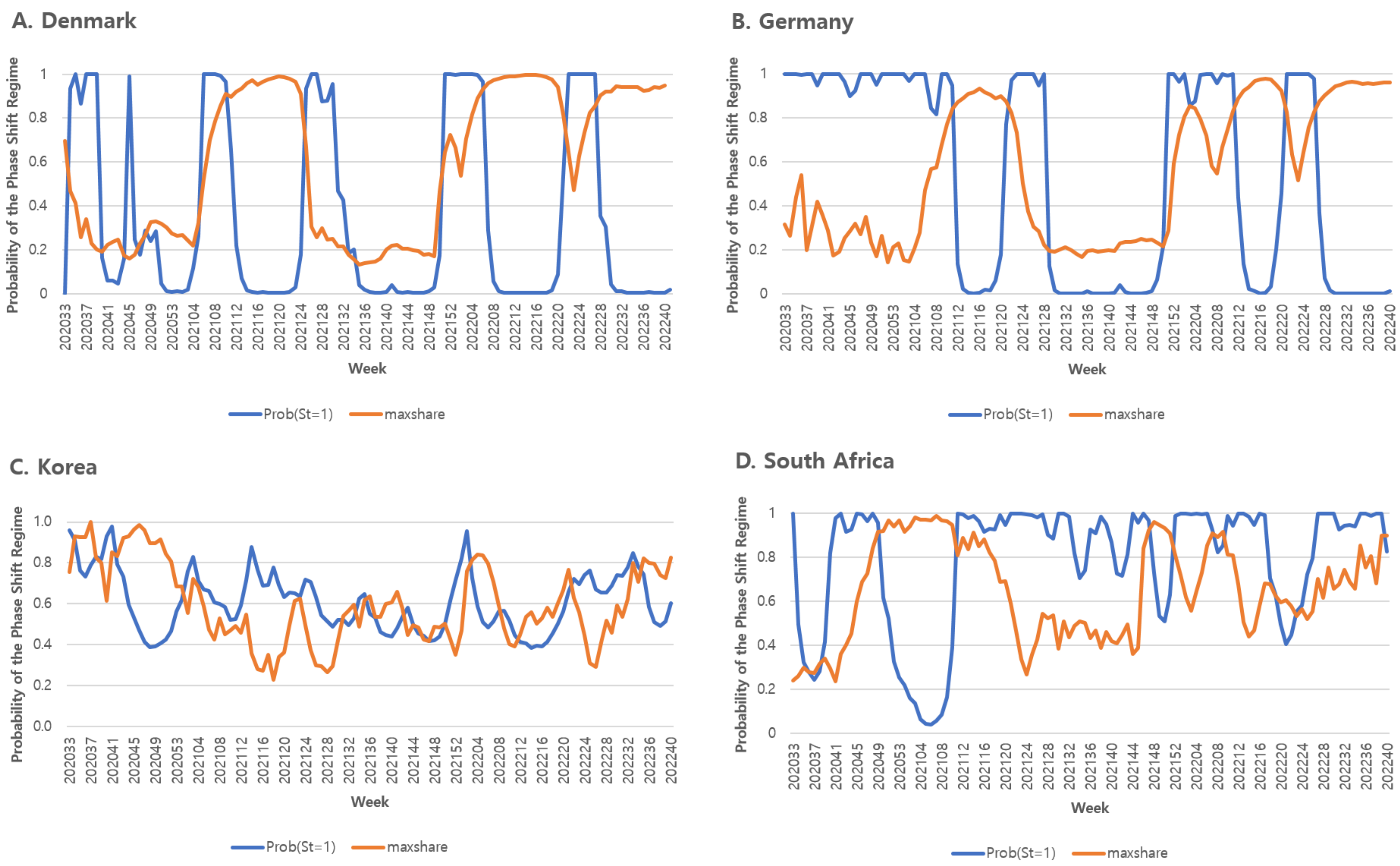

E. United Kingdom

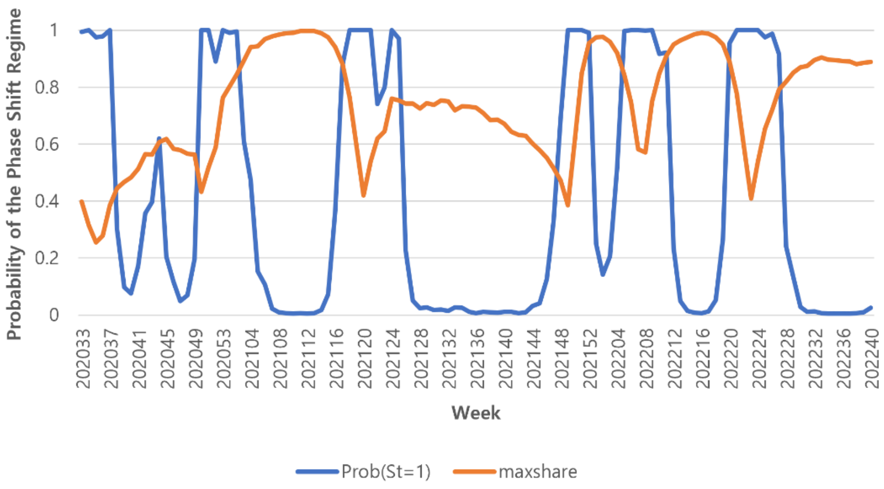

F. United States

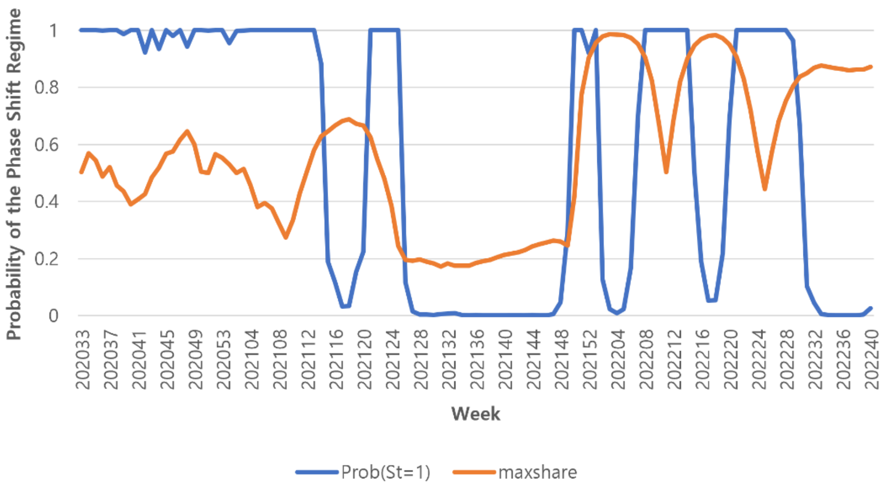

G. Worldwide

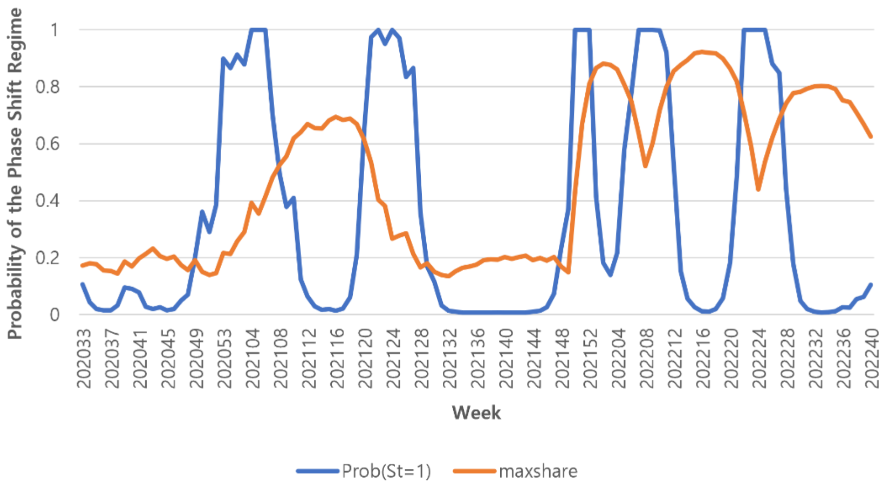

Supplementary References

1. Hansen, Bruce E. Approximate Asymptotic P Values for Structural-Change Tests. 1997. Journal of Business & Economic Statistics, 15(1): 60–67. doi; 10.2307/1392074.
